# Typical Heterotrophic and Autotrophic Nitrogen Removal Process Coupled with Membrane Bioreactor: Comparison of Fouling Behavior and Characterization

**DOI:** 10.3390/membranes14100214

**Published:** 2024-10-07

**Authors:** Qiushan Liu, Tong Zhou, Yuru Liu, Wenjun Wu, Yufei Wang, Guohan Liu, Na Wei, Guangshuo Yin, Jin Guo

**Affiliations:** National Engineering Laboratory for Advanced Municipal Wastewater Treatment and Reuse Technology, Beijing University of Technology, Ping Leyuan No. 100, Beijing 100124, China; liuqiushan@emails.bjut.edu.cn (Q.L.); zhout@emails.bjut.edu.cn (T.Z.); liuyuru@emails.bjut.edu.cn (Y.L.); wuwenjun@emails.bjut.edu.cn (W.W.); wangyufei@emails.bjut.edu.cn (Y.W.); liuguohan@emails.bjut.edu.cn (G.L.); weina@emails.bjut.edu.cn (N.W.); yinguangshuo@emails.bjut.edu.cn (G.Y.)

**Keywords:** partial nitritation–anammox nitrogen removal process, anoxic–oxic nitrogen removal process, membrane bioreactor, membrane fouling, microbial community

## Abstract

There is limited research on the relationship between membrane fouling and microbial metabolites in the nitrogen removal process coupled with membrane bioreactors (MBRs). In this study, we compared anoxic-oxic (AO) and partial nitritation–anammox (PNA), which were selected as representative heterotrophic and autotrophic biological nitrogen removal–coupled MBR processes for their fouling behavior. At the same nitrogen loading rate of 100 mg/L and mixed liquor suspended solids (MLSS) concentration of 4000 mg/L, PNA-MBR exhibited more severe membrane fouling compared to AO-MBR, as evidenced by monitoring changes in transmembrane pressure (TMP). In the autotrophic nitrogen removal process, without added organic carbon, the supernatant of PNA-MBR had higher concentrations of protein, polysaccharides, and low-molecular-weight humic substances, leading to a rapid flux decline. Extracellular polymeric substances (EPS) extracted from suspended sludge and cake sludge in PNA-MBR also contributed to more severe membrane fouling than in AO-MBR. The EPS subfractions of PNA-MBR exhibited looser secondary structures in protein and stronger surface hydrophobicity, particularly in the cake sludge, which contained higher contents of humic substances with lower molecular weights. The higher abundances of *Candidatus* Brocadia and *Chloroflexi* in PNA-MBR could lead to the production of more hydrophobic organics and humic substances. Hydrophobic metabolism products as well as anammox bacteria were deposited on the hydrophobic membrane surface and formed serious fouling. Therefore, hydrophilic membrane modification is more urgently needed to mitigate membrane fouling when running PNA–MBR than AO–MBR.

## 1. Introduction

Discharging untreated nitrogen-containing wastewater into water bodies can lead to severe eutrophication, harming the aquatic ecosystems and human health. The biological nitrogen removal process has been recognized as an effective technology to reduce eutrophication. The biological nitrogen removal process includes heterotrophic and autotrophic biological nitrogen removal processes. Typical heterotrophic biological nitrogen removal technology, such as aerobic nitrification and anaerobic denitrification, has been widely used in conventional wastewater treatment plants. However, these processes are characterized by high energy consumption, extensive space requirements, and the generation of substantial sludge [1]. Compared with the heterotrophic process, the autotrophic nitrogen removal process significantly decreases the need for carbon sources, minimizes sludge production, and reduces aeration consumption [2]. However, the long proliferation rate of functional bacteria in the autotrophic nitrogen removal process usually results in a long period of start-up [2,3].

Membrane biological reactors (MBRs) have been widely used in industrial and domestic wastewater treatment processes in recent years. The MBR process not only achieves complete separation of solids retention time (SRT) and hydraulic retention time (HRT) but also improves the biomass retention of functional bacteria and reduces the ecological footprint [2,4]. Currently, many studies have reported the successful application of both autotrophic and heterotrophic nitrogen removal processes in MBR [2,5,6,7]. However, membrane fouling remains a non-negligible problem during the operation of the MBR process. The presence of membrane fouling reduces membrane flux and increases operating costs, further limiting the widespread application of MBR. Generally, dissolved organic matter (DOM), soluble microbial products (SMP), and extracellular polymeric substances (EPSs) are the main substances contributing to membrane fouling [8,9]. These organic foulants, including polysaccharides, proteins, humic substances, etc. [10], could clog the pores and form a cake layer, resulting in the decline of flux [4].

Substantial efforts have been made to investigate the fouling mechanisms occurring in the heterotrophic nitrogen removal MBR process, such as the use of aerobic membrane bioreactors. Hence, membrane fouling in the heterotrophic nitrogen removal MBR process is relatively well understood [5,6]. Generally, the understanding of fouling and its control methods in the autotrophic nitrogen removal MBR process has been extrapolated from past studies on the heterotrophic nitrogen removal MBR process. However, this may not be appropriate, as heterotrophic nitrogen removal processes use nitrification and denitrification to remove NH_4_^+^-N, whereas autotrophic nitrogen removal processes convert NH_4_^+^-N in the absence of an external carbon source. Therefore, heterotrophic and autotrophic denitrification MBR processes encompass different nitrogen removal mechanisms, distinct microbial communities, and EPSs. For instance, Zheng et al. [11] found that in the autotrophic nitrogen removal process coupled with MBR, microbial metabolites were the main foulants due to the lack of external organic carbon. Ni et al. [12] discovered that anammox bacteria secrete more EPSs than anaerobic and aerobic bacteria. Hou et al. [13] indicated that a larger number of hydrophobic functional groups has been reported in the EPSs of anammox sludge compared to nitrifying and denitrifying sludge. To date, only a few studies have focused on membrane fouling in the autotrophic nitrogen removal MBR process [3,10], with the majority of research concentrating on bacterial aggregation [14,15], the enrichment of microbial communities [16,17], and reactor performance [18,19]. There is a notable lack of attention paid to membrane fouling, and no comparisons have been conducted between heterotrophic and autotrophic nitrogen removal MBR processes.

Therefore, in this study, anoxic–oxic (AO) and partial nitritation–anammox (PNA) were selected as representative heterotrophic and autotrophic biological nitrogen removal processes. By coupling these nitrogen removal processes with MBR under the same nitrogen loading rates, a comprehensive investigation and comparison of the fouling behaviors in the two reactors were conducted. The aims of this study include (1) clarifying the membrane fouling discrepancies between AO-MBR and PNA-MBR; (2) investigating the relationship between membrane fouling and microbial metabolites in AO-MBR and PNA-MBR; and (3) analyzing the relationship between membrane fouling and microbial communities in AO-MBR and PNA-MBR. This study provided a significant theoretical foundation for effective fouling control in nitrogen removal processes coupled with MBR.

## 2. Materials and Methods

### 2.1. Model of Biological Nitrogen Removal–Coupled MBR Reactor

AO-MBR and PNA-MBR had the same working volume of 18L and used continuous flow reactors (Figure S1). The AO-MBR was rectangular (L 600 mm × W 150 mm × H 300 mm), while the PNA-MBR was cylindrical (ID: 200 mm × H 750 mm). Sludges were collected from two full-scale wastewater treatment plants (WWTPs) in Beijing, which, respectively, used PNA and A^2^O biological treatment processes. After removing inorganic impurities and diluting the mixed liquor suspended solids (MLSS) to 4000 mg/L, the sludge was supplied to the MBR. The AO-MBR was successfully initiated after a 5-day acclimatization period, while the PNA-MBR required a 30-day acclimatization period before initiation. Subsequently, the membrane module was placed into the reactor, and formal experiments commenced. A baffle was arranged between the main body of the reactor and the membrane surface in the AO-MBR to prevent direct contact between the aeration device and the membrane. The submerged hollow fiber polyvinylidene fluoride (PVDF) membrane modules (Hai Ke, Guangzhou, China) were installed with an average membrane pore size of 0.1 µm and an effective filtration area of 0.1m^2^. To ensure consistent operational conditions, a temperature controller was utilized to maintain the temperature at 30 ± 2 °C.

Both reactors were fed with synthetic wastewater, with composition details provided in Text S1. The nitrogen loading rate was controlled at 100 mg N/L for both reactors, and the HRT was 1.8 days. The SRT was approximately 40 days for the AO-MBR and 60 days for the PNA-MBR to maintain the same MLSS. Differences in influents between the two reactors were due to the presence of an added carbon source and variations in alkalinity. Aerobic conditions (Dissolved Oxygen about 2 mg/L) and microaerobic environments (Dissolved Oxygen < 0.1 mg/L) were maintained in the AO-MBR and PNA-MBR, respectively. According to other studies [10,20], the effluent was discharged through intermittent membrane suction, operating for 8 min and then stopping for 2 min. The transmembrane pressure (TMP) of the MBR was monitored with a pressure transducer connected to a Programmable Logic Controller (PLC) center and a computer. The filtration process ended when the TMP exceeded 35 kPa, and a ten-minute backwash was performed after each filtration cycle.

### 2.2. Sample Collection

After a 5-day acclimatization period, samples were taken from the AO-MBR, whereas samples from the PNA-MBR were obtained after a stable acclimatization period of 30 days. Both reactors were in a steady state at the time of sampling, with an MLSS concentration of approximately 4000 mg/L. The supernatant was obtained by filtering the activated sludge mixture in reactors through a 0.45 µm PES membrane filter (Tianjin Dongtang Technology Co., Ltd., Tianjin, China). The supernatant of AO-MBR was taken from the aerobic tank where the membrane module was located. The supernatant of the PNA-MBR was taken from the upper part of the reactor. The effluents of both MBRs were collected for further analysis.

A modified heat method [21] was used to extract the EPS subfractions from suspended sludge and cake sludge. The activated sludge was taken from the mixed liquor near the membrane module, and the cake sludge was physically scraped and rinsed off with deionized water from the fouled membrane surface. Briefly, the sludge suspension was initially centrifuged at 4000× *g* for 5 min in a 50 mL tube to dewater it. After filtration through a 0.45 μm membrane, SMP was obtained. The sludge was then diluted back to its original volume of 50 mL using a pre-heated 70 °C NaCl solution. The sludge was sheared for 1 min using a vortex mixer, followed by centrifugation at 4000× *g* for 10 min. The organic matter in the supernatant was considered loosely bound EPS (LB-EPS). The remaining sludge in the centrifuge tube was resuspended in 50 mL of 0.05% NaCl solution. The sludge suspension was heated to 60 °C in a water bath for 30 min then centrifuged at 4000 g for 15 min. The supernatant, filtered through a 0.45 μm membrane, was regarded as tightly bound EPS (TB-EPS).

### 2.3. Analytical Methods

Protein (PN) content was determined according to the modified Lowry method [22], while the polysaccharide (PS) content was obtained using the phenol–sulfuric acid method [23]. The fluorescence spectra of the three-dimensional excitation (Ex)—emission (Em) matrix were obtained using a fluorescence spectrometer (F-7000, Hitachi, Tokyo, Japan) at a scanning speed of 2400 nm/min. The Ex-wavelength ranged from 200 to 400 nm at 5 nm intervals, and the Em-wavelength ranged from 200 to 550 nm with a 5 nm sampling interval. The Ex and Em slit bandwidths were both set at 5 nm. For Fourier-transform infrared spectrometer (FTIR) analysis, collected solutions (10mL) were lyophilized and dried at −80 °C for 72 h. The powder was subsequently blended with KBr at a ratio of 1:100. The mixture was then compressed and analyzed using an FTIR spectrometer (Thermo Scientific, Waltham, MA, USA) across the range of 4000 cm^−1^ to 400 cm^−1^. Furthermore, the secondary structure of the protein was investigated by PeakFit (version 4.12).

The molecular weights (MW) of collected samples were determined using a size-exclusion chromatography (SEC) unit (Shimadzu Prominence, Kyoto, Japan), equipped with an SEC column (TSKgel G3000SW_xL_) and a mobile phase of 5N phosphoric acid + 240 mM phosphate buffer. The samples were filtrated through a 0.22 μm membrane and then carried out using a UV_254_ detector. The TOC detector quantifies TOC concentration by UV oxidation. Polystyrene sulfonate (PSS) standards (4230, 6520, 9680, 14,900, and 29,100 Da) were used as standard molecular weights to convert the retention time to MW.

The surface morphology of the membrane surface was analyzed by a scanning electron microscopy analyzer (SEM, SU9000, Hitachi, Tokyo, Japan). In brief, the membrane fiber was first fixed in 2.5% glutaraldehyde for 10 h. Then, the membrane was dewatered by immersion in a series of ethanol solutions of 50%, 70%, 80%, 90%, and 100%, for 15min at each ethanol concentration. The obtained samples were sputter-coated with gold palladium before the membrane was examined under the SEM.

Standard methods were used to determine the concentrations of NH_4_^+^-N, NO^2−^-N, and NO^3−^-N [24]. The dissolved organic carbon (DOC) was measured using a total organic carbon (TOC) analyzer (TOC-L CSN, Shimadzu, Kyoto, Japan). UV_254_ was obtained using a UV–visible spectrophotometer (P1, MAPADA, Shanghai, China). The pH was measured using multiparameter water quality instruments (WTW Multi 3620 IDS, Xylem Analytics, Mainz, Germany). All the analyses were performed three times to obtain the average value.

### 2.4. Dead-End Membrane Filtration Experiment

The samples (aqueous pollutants and EPS subfractions) were subjected to dead-end filtration mode under a pressure of 1 bar. Polyvinylidene fluoride (PVDF) membranes (Zhongli Filtration Co., Ltd., Haining, China) with a pore size of 0.1 μm and an effective area of 11.3 cm^2^ were used to evaluate membrane fouling. The pure water flux for sheet membranes at 1 bar pressure was around 2500 ± 10% LMH. The dead-end filtration mode and the calculation of the normalized flux and the membrane fouling resistance are shown in Figure S2.

### 2.5. Microbial Community

The microbial community of the suspended sludge and the cake sludge was performed using Illumina high-throughput sequencing. This sequencing was conducted by Shanghai Majorbio (Shanghai, China). Specifically, the amplification of the V3–V4 region of the 16S rRNA gene was carried out using bacterial primers 338F (5′-ACT CCT ACG GGA GGC AGC AG-3′) and 806R (5′GGA CTA CHV GGG TWT CTA AT-3′). The data were processed and analyzed on the Majorbio Cloud Platform.

## 3. Results and Discussion

### 3.1. Nitrogen Removal and the Temporal Variation of TMP in AO-MBR and PNA-MBR

**Figure 1:**
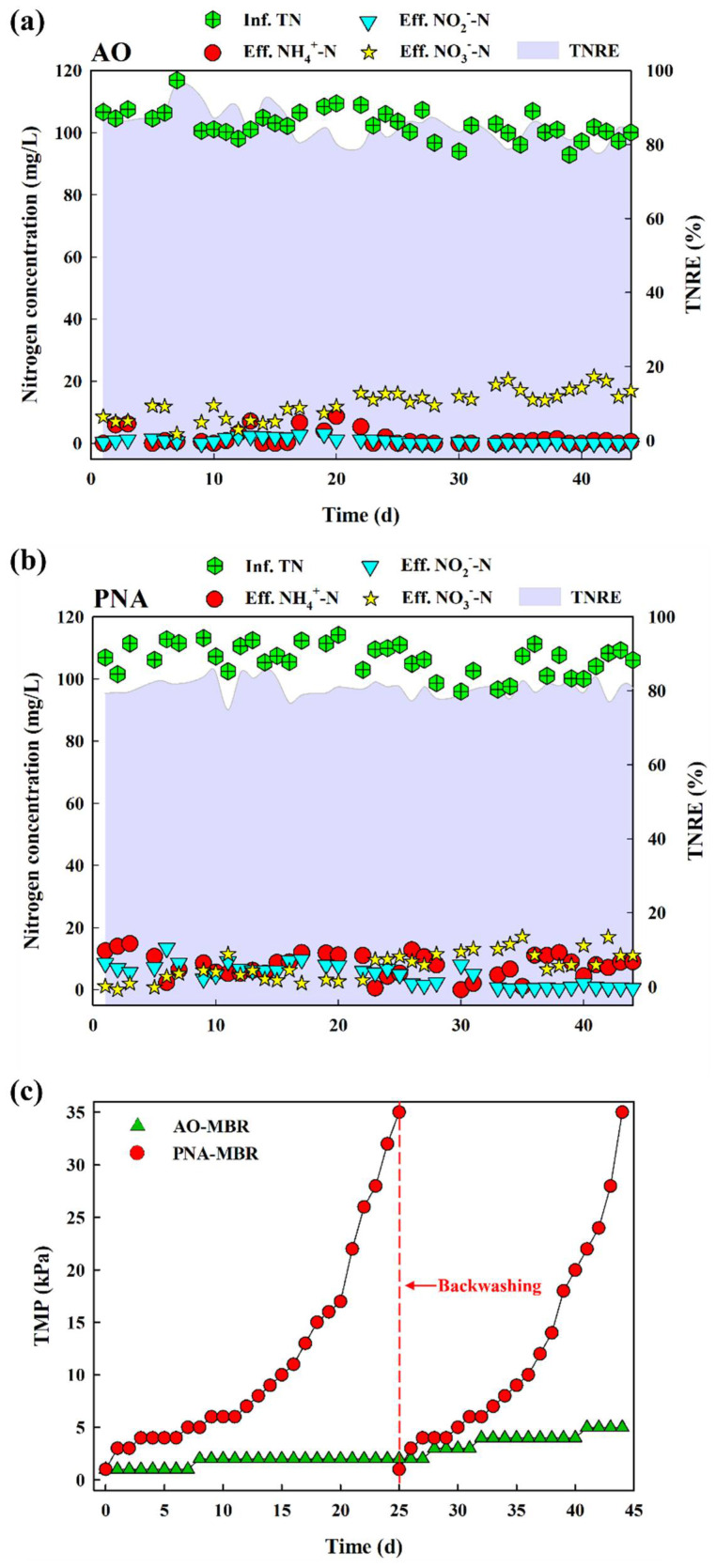
The concentration of NH_4_^+^−N, NO_2_^−^−N, and NO_3_^−^ − N in the influent and effluent of (**a**) AO−MBR and (**b**) PNA−MBR and the temporal variation in TMP during the operation of the two reactors (**c**).

After the reactor reached steady-state operation, formal experiments commenced. As shown in Figure 1a, during the whole operation period (44 days), AO-MBR exhibited a fairly stable nitrogen removal performance, with the concentrations of NH_4_^+^-N (1.57 ± 2.39 mg/L), NO_2_^−^-N (0.79 ± 0.86 mg/L), and NO_3_^−^-N (12.77 ± 4.57 mg/L) in the effluent. In this process, NH_4_^+^-N was converted to NO_3_^−^-N by nitrification and then to N_2_ by denitrification. At the same time, total nitrogen removal efficiency (TNRE) was 85.12 ± 4.21%, indicating effective nitrogen removal performance by the AO-MBR. In PNA-MBR (Figure 1b), NH_4_^+^-N was first converted to NO_2_^−^-N by partial nitration. Subsequently, NH_4_^+^-N and NO_2_^−^-N were converted to NO_3_^−^-N and N_2_ by anammox [2]. The TNRE was 80.87 ± 2.42%, with the concentration of NH_4_^+^-N, NO_2_^—^N, and NO_3_^−^-N in the effluent being 7.81 ± 3.77 mg/L, 4.68 ± 3.43 mg/L, and 7.75 ± 4.54 mg/L, respectively. Although the nitrogen removal performance of the two reactors was similar, their TMP temporal variation showed significant differences**.**

Figure 1c shows the temporal variation in TMP during the operation of the two reactors. The constant permeate flux J in the MBR was approximately 4 LMH, which is within the range reported in similar literature [3,7,25] (5.88 LMH, 2.42 LMH, and 1.89 LMH). PNA–MBR exhibited more serious fouling (35 kPa on day 25) compared to the AO–MBR (2 kPa on day 25). After backwashing, the second filtration cycle of PNA-MBR continued for 19 days, which was shorter than the first filtration cycle. The TMP of the AO-MBR only increased to 5 kPa after the 44-day operation. SEM (Figure S3) showed the membrane surface morphologies before and after foulant deposition. Compared to the virgin membrane, both the PNA–MBR and AO-MBR exhibited significant fouling deposition on the membrane surface. Nevertheless, PNA–MBR exhibited a more pronounced presence of gel-like substances, which is suspected to be associated with its more severe membrane fouling. As depicted in Figure S4, one of the two filtered samples consisted of a sludge mixture, while the other comprised dissolved organic matter obtained by filtering the sludge mixture through a 0.45 μm membrane. The dissolved organic matter obtained after 0.45 μm membrane filtration represented organic fouling, while the sludge mixture represented total fouling. It could be observed that membrane fouling caused by filtration of dissolved organic matter was alleviated compared to filtration of the sludge mixture. In comparison to AO-MBR, membrane fouling was less alleviated in PNA-MBR, indicating that organic fouling exerted a more pronounced effect on membrane fouling in PNA-MBR. During the operation of both reactors, the sludge mixture and dissolved organic matter were filtrated using a dead-end unit, respectively. Evidently, the sludge mixture from AO-MBR resulted in more serious fouling, while its fouling was greatly alleviated by filtering through the 0.45 µm membrane. Conversely, fouling caused by the activated sludge mixture from PNA-MBR could not be alleviated by filtering through the 0.45 µm membrane, which suggested that the dissolved organic pollutants in the activated sludge mixture of PNA-MBR played a more significant role in fouling formation.

To further elucidate their different fouling behaviors, the supernatant, suspended sludge, and cake sludge of both PNA-MBR and AO-MBR were separately evaluated for their corresponding membrane fouling.

### 3.2. Fouling Behavior and Characterization of Supernatant

**Figure 2:**
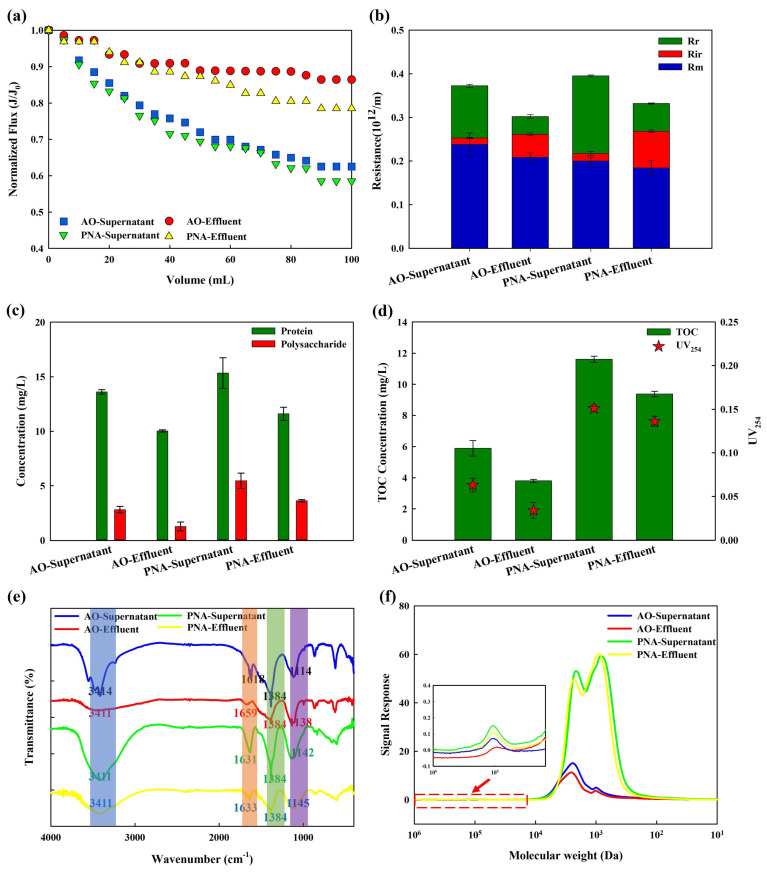
Fouling behavior and characterization of AO/PNA supernatant and effluent of AO/PNA−MBR. (**a**) Normalized flux transformation; (**b**) membrane resistance calculation; (**c**) protein and polysaccharide concentration; (**d**) total organic carbon (TOC) and UV_254_; (**e**) FTIR analysis; (**f**) MW distribution.

The fouling behavior of supernatants in AO-MBR and PNA-MBR was further analyzed via a dead-end filtration experiment. The normalized flux transformation is demonstrated in Figure 2a. The supernatant in PNA-MBR caused more severe membrane fouling than AO-MBR. In addition, membrane fouling caused by the effluent of both PNA-MBR and AO-MBR was alleviated compared with their supernatant. The reversibility of membrane fouling is also analyzed in Figure 2b. The total membrane fouling resistance (R_t_) caused by the supernatant and effluent of PNA-MBR was all higher than that of AO-MBR, as well as the reversible membrane fouling resistance (R_r_) and irreversible membrane fouling resistance (R_ir_). In both reactors, the supernatant was primarily composed of polysaccharides and proteins. The concentrations of protein and polysaccharide are shown in Figure 2c. PNA-MBR had higher concentrations of proteins and polysaccharides (15.3 ± 1.4 mg/L and 5.5 ± 0.7 mg/L in supernatant, 11.6 ± 0.6 mg/L and 3.6 ± 0.1 mg/L in effluent) compared to AO-MBR (13.6 ± 0.2 mg/L and 2.8 ± 0.3 mg/L in supernatant, 10.0 ± 0.1 mg/L and 1.3 ± 0.4 mg/L in effluent). As reported, the non-covalent network formed by protein and polysaccharides might improve the membrane fouling potential [26,27]. Figure 2d shows the concentration of TOC and UV_254_. Without an external organic carbon source, PNA-MBR exhibited higher TOC and UV_254_ both in the supernatant (11.6 ± 0.2 mg C/L and 0.15 ± 0.004) and effluent (9.4 ± 0.2 mg C/L and 0.14 ± 0.006) than the AO-MBR. The supernatant in PNA-MBR had higher aromaticity which might relate to the presence of lignin, humic acid, aromatic proteins, etc. [28]. Zhou et al. (2024) also found that PNA produced more aromatic DOM than A^2^O in full-scale wastewater treatment plants [29]. The EEM results of the supernatant are demonstrated in Figure S5. The peaks detected at excitation/emission wavelengths of 200–275/380–520 nm and 275–400/380–520 nm were generally identified as fulvic acid and humic-like substances [7,30]. The emission wavelengths of humic substances differed slightly between PNA-MBR (260 nm) and AO-MBR (255 nm), indicating differences in the supernatant. The fluorescence intensity in the supernatant of PNA-MBR was much higher than that of AO-MBR, indicating a higher concentration of humic and fulvic substances in PNA-MBR.

To further confirm the different properties of these aqueous pollutants, FTIR analyses were carried out. In the FTIR spectrum (Figure 2e), the peak at 3411 cm^−1^ was attributed to the stretching vibrations of N-H and O-H in protein and polysaccharides [3], and 1600 cm^−1^ to 1700 cm^−1^ was linked to the protein-like substances [31]. Carboxylic groups and hydrocarbon-like compounds occurred at 1500–1300 cm^−1^ [32]. The range of 1114–1145 cm^−1^ represented C-O-C stretching vibrations in polysaccharides [31]. The FTIR spectra of the supernatant in both AO-MBR and PNA-MBR exhibited similar peaks and distributions, indicating the presence of comparable functional groups. However, different transmittance rates suggest variations in the structural composition of the supernatants from PNA-MBR and AO-MBR. This result was consistent with the protein and polysaccharide concentrations shown in Figure 2c, as well as the TOC in Figure 2d. Figure 2f shows the molecular weight distribution (MWD) of the supernatant. The major absorbance peaks with a high UV response were characterized as humic substances, while the biopolymers displayed a relatively weak UV response [33]. It could be observed that the response of biopolymer and humic substances in the supernatant and effluent of PNA-MBR were all significantly higher than AO-MBR, and more low-MW humic substances (~1000 Da) were presented in PNA-MBR. Humic substances were highly hydrophobic and could bind to polysaccharides and protein due to hydrophobic and electrostatic interactions [34]. When humic substances adhere to the membrane surface and block the membrane pores, it becomes more probable for other hydrophilic compounds, like polysaccharides, to accumulate on the membrane surface resulting in severe membrane fouling [35]. The higher concentration of low-MW humic substances in PNA-MBR might be one of the reasons for its severe membrane fouling.

### 3.3. Fouling Behavior and Characterization of Suspended Sludge and Cake Sludge

#### 3.3.1. Membrane Fouling Behavior

**Figure 3:**
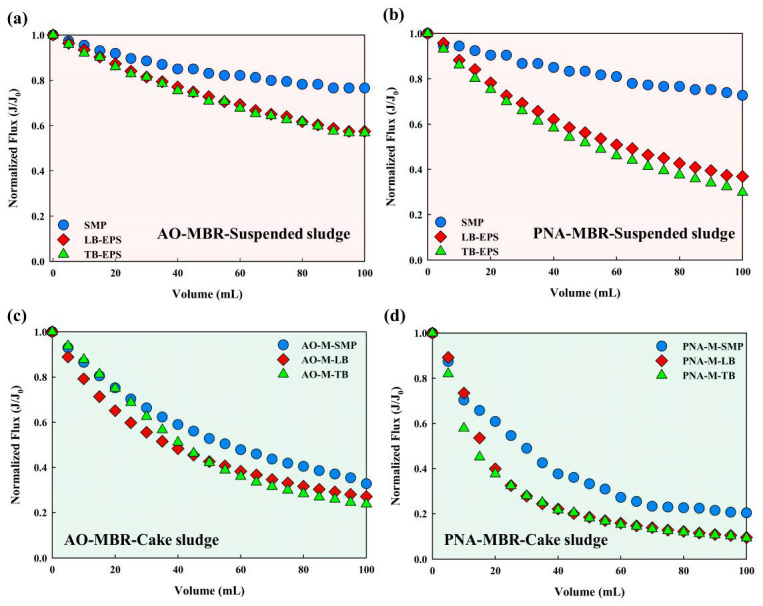
Normalized flux transformation. (**a**,**b**) EPS extracted from the suspended sludge; (**c**,**d**) EPS extracted from the cake sludge.

Investigations have revealed that sludges play a crucial role in the temporal variation of TMP [2,26]. Figure 3 demonstrates the evolution of normalized flux (J/J0) related to suspended sludge and cake sludge. To exclude the interference on membrane fouling by organic carbon concentration, the EPS subfractions extracted from suspended sludge and cake sludge were diluted to the same DOC concentration. Figure 3a,b show the J/J_0_ evolution of EPS subfractions extracted from the suspended sludge, and Figure 3c,d demonstrate that extracted from the cake sludge. Figure 3b indicates that the final normalized flux of SMP, LB-EPS, and TB-EPS in PNA-MBR was 0.72, 0.37, and 0.30, respectively, showing a faster flux decline compared to AO-MBR (Figure 3a). Similarly, Figure 3d also illustrates a faster flux decline for the EPS subfractions extracted from cake sludge of PNA-MBR. Regardless of whether the sludge is suspended or cake sludge in PNA-MBR, the EPS subfractions consistently lead to more severe membrane fouling. The reversibility of membrane fouling is analyzed in Figure S6, which shows that the R_t_ and R_ir_ caused by the EPS subfractions extracted from PNA-MBR were all higher than that in AO-MBR.

#### 3.3.2. Characterization of Suspended Sludge and Cake Sludge

**Figure 4:**
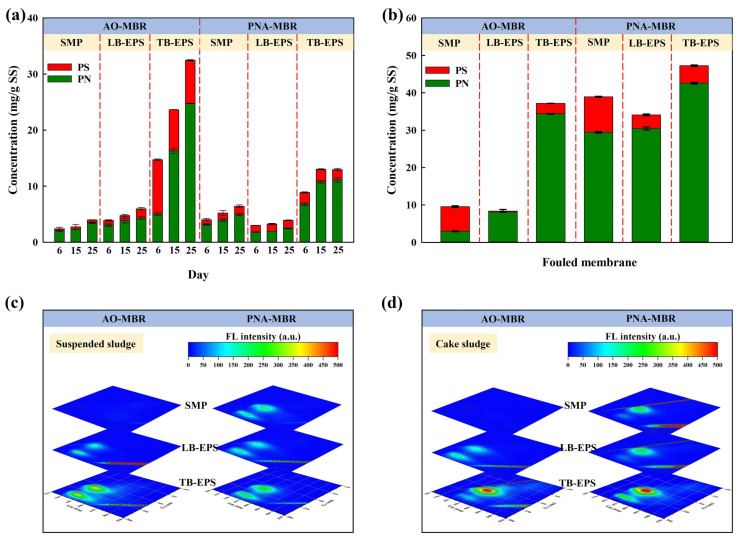
Contents and EEM analyses of SMP, LB-EPS, and TB-EPS in suspended sludge (**a**,**c**) and the cake sludge on day 44 (**b**,**d**) in the AO-MBR and PNA-MBR.

As shown in Figure 4a, there was a noticeable increase in the concentrations of protein and polysaccharides in the suspended sludge of both reactors during the operational phase. The EPS subfractions extracted from the suspended sludge in AO-MBR had higher concentrations than those from PNA-MBR, except for SMP. As reported, the aqueous pollutants in MBR were normally produced by the SMP [26] and a slimy and gel-like layer with low permeability and high filtration resistance was formed on the membrane surface due to the accumulation of SMP [2,36]. The short proliferation rate and high metabolic rate of heterotrophic bacteria in AO-MBR would increase the biomass amount and accumulate the SMP [37,38]. Nevertheless, in our experiment, the MLSS concentration of both reactors was controlled at 4000 mg/L by irregularly discharging suspended sludge. Furthermore, under a lower nitrogen loading (100 mg N/L), the PNA-MBR might produce more SMP during the adaptation process of functional microbiota, which could contribute to severe membrane fouling. After performing three tests to obtain the average values, Figure 4b shows that the content of EPS extracted from the cake sludge of PNA-MBR was all higher than that of AO-MBR. In Figure 4c,d, the EEM of EPS subfractions revealed two major fluorescence peaks. The peaks at the Ex/Em of 250–280/280–380 nm and 200–250/280–380 nm were correlated with tryptophan-like protein and tyrosine-like protein, respectively [3,39]. The characteristic peaks of EPS in AO-MBR were blue-shifted by 5-10 nm compared to those of PNA-MBR. The shifts in peak locations indicated that the structure of protein-like substances differed in the two MBRs, which might also lead to differences in membrane fouling behaviors. Therefore, the differences in the secondary structure of proteins from the EPS subfractions were further analyzed.

#### 3.3.3. The Protein Secondary Structure of Suspended Sludge and Cake Sludge

The secondary structure of proteins has been reported to strongly influence the hydrophobicity of sludge [3,15]. Therefore, the FTIR spectra (1600–1700 cm^−1^ range) of EPS extracted from suspended sludge and cake sludge were examined. The fitted curves are shown in Figures S8 and S9, and Table S2 lists the relative concentrations of α-helix, β-sheet, β-turn, and random coil as well as the ratio of α-helix/(β-sheet+ random coil).

Previous studies have shown that a higher content of β-sheet in protein might contribute to the increased dispersion of microorganisms [40]. The ratio of α-helix/(β-sheet + random coil) could provide an insight into protein structure, with lower ratios typically indicating a looser protein structure and stronger hydrophobicity [15]. As shown in Table S2, after three measurements to obtain an average value, the EPS subfractions extracted from suspended sludge of PNA-MBR had a looser structure compared to AO-MBR, with lower α-helix/(β-sheet + random coil) values of 0.25, 0.17, and 0.15. This indicated that the protein in the suspended sludge of PNA-MBR had stronger hydrophobicity than that in AO-MBR. Some studies have concluded that higher hydrophobicity usually leads to large flocs and reduced membrane fouling [41]. Nevertheless, as for the EPS subfractions extracted from the cake sludge, the ratio of α-helix/(β-sheet+ random coil) in PNA-MBR was also lower than in AO-MBR. These findings revealed that the EPS extracted from both suspended sludge and cake sludge in PNA-MBR all had looser protein structures and stronger surface hydrophobicity. Hou et al. [13] also noted that the EPS of anammox sludge contained a higher quantity of hydrophobic functional groups compared to nitrifying and denitrifying sludge.

#### 3.3.4. Molecular Weight Distribution

Figure S10 displayed the MWD analysis of EPS subfractions extracted from suspended sludge and cake sludge of AO-MBR and PNA-MBR. The chromatograms were divided into biopolymer and humic substances. The humic substances were further categorized into humic-like acids, fulvic-like acids, building blocks, and low molecular weight neutral substances [42]. There existed an irregular discrepancy of concentration for the EPS subfractions extracted from the suspended sludge and cake sludge, as shown in Figure S10a,b. Nevertheless, lower MW of humic substances was presented in the EPS subfractions extracted from PNA-MBR, especially in the cake sludge. This corresponded to the MWD of aqueous pollutants in PNA-MBR (Figure 2f). As demonstrated in Figure S10b, the SMP, LB-EPS, and TB-EPS extracted from the cake sludge all showed pronounced differences in MWD of humic substances between the PNA-MBR and AO-MBR. Overall, as displayed in Figure S10d, the EPS subfractions extracted from PNA-MBR contained more humic substances with lower MW of <550 Da.

### 3.4. Microbial Community Comparison between AO-MBR and PNA-MBR

**Figure 5:**
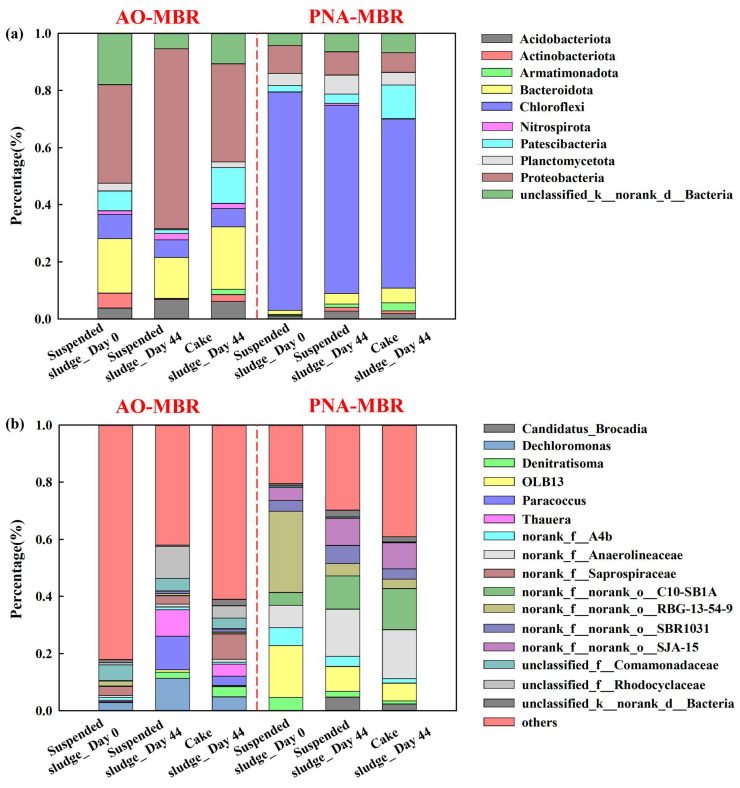
Taxonomic distribution of the microbial community in the sludge of the AO-MBR and PNA-MBR in the operational phase (0 d and 44 d) of the suspended sludge and the cake sludge at phylum (**a**) and genus (**b**) level by 16S rRNA sequencing.

Figure 5 and Figures S11–S14 illustrate the relative microbial abundances of suspended sludge and cake sludge in AO-MBR and PNA-MBR at the phylum and genus levels. Figure 5a shows the phylum level in AO-MBR, where *Proteobacteria* and *Bacteroidetes* were the dominant phyla. After 44 days of operation, the abundance of *Proteobacteria* increased from 35% to 63%, while the abundance of *Bacteroidota* decreased from 19% to 14%. *Proteobacteria*, the dominant phylum, played a crucial role in denitrification and the degradation of diverse pollutants in wastewater treatment [43]. *Bacteroidetes*, a significant class of heterotrophic organisms, played a significant role in the recycling of organic carbon and proteins [43]. These indicated that AO-MBR performed well throughout the operational period. As shown in Figure 5a, *Chloroflexi*, *Proteobacteria,* and *Planctomycetes* were identified as the primary microorganisms in suspended sludge for PNA-MBR, which was similar to the microbial community found in another PNA system [18]. In particular, during the operation, the relative abundance of *Chloroflexi* decreased from 76% to 66%, the *Proteobacteria* decreased from 10% to 8%, and *Planctomycetes* increased from 4% to 7%. As reported, these phyla were widely distributed in the anammox reactors and played essential roles in the anammox process [3]. In wastewater treatment, *Chloroflexi* bacteria often coexist with anammox bacteria, relying on the degradation of protein secreted by anammox [44]. At the same time, *Chloroflexi* has the capability to ferment sugars and organic compounds originating from anammox bacteria [45]. Moreover, this phylum could promote the dissipation and humification of organic pollutants [45], leading to higher humic acid content and smaller molecular weight of the metabolites in PNA-MBR (Figure 2f and Figure S10). *Proteobacteria* has been observed the ability to transfer the electrons directly to the electrode and *Planctomycetes* can oxidize NH_4_^+^-N in the absence of molecular oxygen, utilizing NO_2_^−^-N as the electron acceptor [46]. These results indicated the enrichment of anammox bacteria in the PNA-MBR. It could be observed that the abundance of *Proteobacteria* and *Chloroflexi* was significantly different between AO-MBR and PNA-MBR (Figure S11a). A higher relative abundance of *Proteobacteria* in AO-MBR promoted the utilization of membrane foulants and the elimination of pollutants [46]. A lower abundance of *Chloroflexi* in AO-MBR might lead to lower humic content than in PNA-MBR. Therefore, the membrane fouling behavior of AO-MBR might have less membrane fouling behaviors than PNA-MBR. The taxonomic compositions and the relative abundance of the microbial community in cake sludge and suspended sludge were similar except for *Patescibacteria* (Figure S12). As shown in Figure 5a, the abundance of *Patescibacteria* in cake sludge was higher than that in suspended sludge both in AO-MBR (13% and 1%) and PNA-MBR (12% and 3%) on day 44. As reported, *Patescibacteria* had the ability to metabolize carbohydrates, amino acids, and other substances adhered to the membrane surface [47]. Therefore, *Patescibacteria* might be more likely to be enriched on membrane surfaces compared to other bacteria. As shown in Figure S11b, the abundance of *Proteobacteria* and *Chloroflexi* was also significantly different in different cake sludge. Further investigation and comparison of these variations were required to gain a deeper understanding of the fouling mechanisms between AO-MBR and PNA-MBR.

The distribution of microbial communities at the genus level in the two reactors was further illustrated in the two reactors was further depicted in Figure 5b, S13, and S14. Figure 5b shows the relative abundance of microorganisms in AO-MBR. After 44 days of operation, the abundance of *Dechloromonas*, *unclassified_f__Rhodocyclaceae*, *Paracoccus,* and *Thauera* increased from 3% to 11%, 1% to 11%, 0% to 12%, and 0% to 9%, respectively. *Dechloromonas* had denitrification and ammonia oxidation functions [43]. *Thauera*, a member of the *Rhodocyclaceae* family, was dominant in wastewater treatment plants, particularly in denitrification units [48]. These indicated that the AO-MBR demonstrated good performance. Three hydrolytic bacteria, i.e., *norank_f__Anaerolineaceae*, *norank_f__norank_o__C10-SB1A,* and *Candidatus_Brocadia*, were found in PNA-MBR (Figure 5b), and their abundances were significantly increased during the operation. *Norank_f__Anaerolineaceae*, a genus of filamentous microorganisms, was dominant in the PNA system, potentially serving as a scaffold for biofilm [18]. The abundance of *norank_f__Anaerolineaceae* increased from 8% on day 0 to 16% on day 44. *C10-SB1A*, a type of hydrolyzer associated with anaerobic carbohydrate degradation [49], increased from 5% to 12%. In suspended sludge, *Candidatus_Brocadia*, a representative anammox bacteria, exhibited stronger aggregation capacity and hydrophobic properties than other bacteria [45] and increased from 0% to 5%. These indicate that MBR could effectively enrich the anammox bacteria. In contrast, the abundance of OLB13 and *Denitratisoma* showed a significant reduction from 18% to 9% and 5% to 2%, respectively. This suggests that the accumulation of NO_2_^-^-N might be reduced [50], and the denitrification process was weakened [44]. Moreover, in the case of cake sludges in AO-MBR, the abundance of *norank_f__Saprospiraceae* and *unclassified_c__Parcubacteria* was higher than that in suspended sludge (Figure S13a). This suggests that these bacteria might adhere more easily to the membrane surface. In the PNA-MBR, *norank_f__norank_o__C10-SB1A* exhibited higher percentages in cake sludge than suspended sludge (Figure S13b), implying a higher potential for biofouling. Figure S14b shows the difference in cake sludge extracted from AO-MBR and PNA-MBR. In PNA-MBR, *norank_f__Anaerolineaceae* and *norank_f__norank_o__C10-SB1A* were higher than that in AO-MBR. Differences in microbial communities on the membrane surface in AO-MBR and PNA-MBR may be an important factor contributing to different membrane fouling characteristics.

These phenomena indicate that PNA-MBR has a higher presence of *Chloroflexi*, which enhances the degradation and transformation of organic pollutants into humic substances. Consequently, there might be a higher concentration of humic substances in the metabolites of PNA-MBR, potentially leading to irreversible membrane fouling. Additionally, the anammox bacteria (*Candidatus* Brocadia) exhibited higher hydrophobicity and adhesion compared to other bacteria, making them more likely to attach to the membrane surface and induce membrane fouling even with lower relative concentrations. These were speculated to be a crucial factor contributing to the different membrane fouling behaviors observed between AO-MBR and PNA-MBR.

### 3.5. Engineering Implications and Limitations

This study explored the differences between AO-MBR and PNA-MBR with a nitrogen loading of 100 mg N /L and MLSS concentrations of approximately 4000 mg/L. However, membrane fouling in both MBR processes might vary with different nitrogen loads and sludge characteristics. Future investigations should test various conditions and employ different detection methods to gain a deeper understanding of membrane fouling. The study indicates that when treated with a nitrogen load concentration of approximately 100 mg/L, AO-MBR causes less membrane fouling compared to PNA-MBR and should be considered preferentially in practical engineering applications. As reported [51], PNA process has been successfully applied in side-stream treatment processes with high NH_4_^+^-N and low C/N ratios, and PNA-MBR also enhances the retention of functional bacteria. Therefore, despite causing more severe membrane fouling in this study, PNA-MBR remains a promising option for treating wastewater with a low C/N ratio. Additionally, the study also found that anammox bacteria as well as hydrophobic metabolism products deposited on the hydrophobic membrane surface and formed serious fouling. Therefore, subsequent studies should focus on hydrophilic membrane modification to explore methods for mitigating membrane fouling in PNA-MBR.

## 4. Conclusions

Under lower nitrogen loading (100 mg N/L) and similar MLSS concentrations (4000mg/L), mainstream PNA-MBR suffers from more severe membrane fouling than AO-MBR. The dead-end filtration experiment also indicated that the flux decline of supernatant and EPS subfractions extracted from the suspended sludge and cake sludge were all faster in PNA-MBR. The discrepancy in membrane fouling was related to the differences in microbial metabolites and microbial communities in the biological nitrogen removal process. The supernatant of PNA-MBR had a higher content of protein (15.3 ± 1.4 mg/L), polysaccharides (5.5 ± 0.7 mg/L), and low-MW humic substances. The EPS subfractions extracted from the suspended sludge and cake sludge in PNA-MBR all had a lower ratio of α-helix/(β-sheet+ random coil), which corresponds to more hydrophobic characteristics. Especially for the cake sludge, a higher content of EPS and lower MW of humic substances was found in PNA-MBR. The microbial communities in AO-MBR and PNA-MBR exhibited significant differences. The abundance of *Candidatus Brocadia* and *Chloroflexi* in PNA-MBR was 5% and 66%, respectively, which was much higher than those in AO-MBR. The microbial metabolites and bacteria in PNA-MBR were more hydrophobic, making them more likely to adhere to the hydrophobic membrane surface, implying a higher membrane fouling potential.

## Data Availability

The datasets supporting the results of this article are included within the article and Supplementary Materials.

## Supplementary Materials

The following supporting information can be downloaded at: https://www.mdpi.com/article/10.3390/membranes14100214/s1, Text 1 Dead-end membrane filtration experiment; Table S1: Ingredient of the synthetic wastewater for AO-MBR and PNA-MBR; Table S2: Relative contents of the protein secondary structures of suspended sludge and cake sludge in AO-MBR and PNA-MBR; Figure S1: Schematic diagram of the AO-MBR (a) and PNA-MBR (b); Figure S2: The schematic diagram of the dead-end filtration experiment; Figure S3: SEM of virgin membrane (a), membrane surface of AO-MBR (c) and PNA-MBR; Figure S4: Normalized flux evolution of activated sludge mixture from AO-MBR (a) and PNA-MBR (b); Figure S5: EEM analysis result of supernatant (a, b) and MBR effluent (c, d) of AO-MBR (a, c) and PNA-MBR (b, d); Figure S6: Membrane resistance of SMP and EPS extracted from AO-MBR and PNA-MBR; Figure S7: FTIR analysis result of SMP, LB-EPS and TB-EPS in active sludge (a, b) and cake sludge (c, d) of AO-MBR (a, c) and PNA-MBR (b, d), respectively; Figure S8: The second derivative resolution-enhanced curve-fitted amide I region (1700–1600 cm−1) of proteins in the active sludge of AO-MBR (a-c) and PNA-MBR (d-f); Figure S9: The second derivative resolution-enhanced curve-fitted amide I region (1700–1600 cm−1) of proteins in cake sludge of AO-MBR (a-c) and PNA-MBR (d-f); Figure S10: Molecular weight distribution of SMP, LB-EPS and TB-EPS in suspended sludge (a, c) and the cake sludges (b, d) of AO-MBR and PNA-MBR; Figure S11: Differences in the phylum level between the suspended sludge (a) and cake sludge (b) in 2 AO-MBR and PNA-MBR determined by the high-throughput sequencing based 16S rRNA genes; Figure S12: Differences of active sludge and cake sludge in the phylum level between AO-MBR (a) and PNA-MBR (b) determined by the high-throughput sequencing based 16S rRNA genes; Figure S13: Differences of active sludge and cake sludge in the genus level between AO-MBR (a) and PNA-MBR (b) determined by the high-throughput sequencing based 16S rRNA genes; Figure S14: Differences in the genus level between the suspended sludge (a) and cake sludge (b) in AO-MBR and PNA-MBR determined by the high-throughput sequencing based 16S rRNA genes.

## References

[1] Abbassi R., Yadav A.K., Huang S., Jaffe P.R. (2014). Laboratory study of nitrification, denitrification and anammox processes in membrane bioreactors considering periodic aeration. J. Environ. Manag..

[2] Chen F., Qian Y., Cheng H., Shen J., Qin Y., Li Y.-Y. (2023). Recent developments in anammox-based membrane bioreactors: A review. Sci. Total Environ..

[3] Cai X., Wang Z., Qian Y., Wang A., Yang Y., Xia S. (2022). Comprehensively understanding fouling properties of cake and bulk sludge in an anammox membrane bioreactor: Focusing on the composition, interfacial thermodynamics and microbial community. J. Environ. Chem. Eng..

[4] Gao D., Fu Y., Ren N. (2013). Tracing biofouling to the structure of the microbial community and its metabolic products: A study of the three-stage MBR process. Water Res..

[5] Chen C.-H., Fu Y., Gao D.-W. (2015). Membrane biofouling process correlated to the microbial community succession in an A/O MBR. Bioresour. Technol..

[6] Gao D.-W., Wen Z.-D., Li B., Liang H. (2014). Microbial community structure characteristics associated membrane fouling in A/O-MBR system. Bioresour. Technol..

[7] Niu Z., Zhang Z., Liu S., Miyoshi T., Matsuyama H., Ni J. (2016). Discrepant membrane fouling of partial nitrification and anammox membrane bioreactor operated at the same nitrogen loading rate. Bioresour. Technol..

[8] Tang S., Wang Z., Wu Z., Zhou Q. (2010). Role of dissolved organic matters (DOM) in membrane fouling of membrane bioreactors for municipal wastewater treatment. J. Hazard. Mater..

[9] Lin H., Zhang M., Wang F., Meng F., Liao B.-Q., Hong H., Chen J., Gao W. (2014). A critical review of extracellular polymeric substances (EPSs) in membrane bioreactors: Characteristics, roles in membrane fouling and control strategies. J. Membr. Sci..

[10] Ni L., Shi Q., Wu M., Ma J., Wang Y. (2021). Fouling behavior and mechanism of hydrophilic modified membrane in anammox membrane bioreactor: Role of gel layer. J. Membr. Sci..

[11] Zheng F., Wang J., Xiao R., Chai W., Xing D., Lu H. (2021). Dissolved organic nitrogen in wastewater treatment processes: Transformation, biosynthesis and ecological impacts. Environ. Pollut..

[12] Ni S.-Q., Sun N., Yang H., Zhang J., Ngo H.H. (2015). Distribution of extracellular polymeric substances in anammox granules and their important roles during anammox granulation. Biochem. Eng. J..

[13] Hou X., Liu S., Zhang Z. (2015). Role of extracellular polymeric substance in determining the high aggregation ability of anammox sludge. Water Res..

[14] Wang W.Y., Wang R., Abbas G., Wang G., Zhao Z.G., Deng L.W., Wang L. (2022). Aggregation enhances the activity and growth rate of anammox bacteria and its mechanisms. Chemosphere.

[15] Wang W., Yan Y., Zhao Y., Shi Q., Wang Y. (2020). Characterization of stratified EPS and their role in the initial adhesion of anammox consortia. Water Res..

[16] Huang X., Mi W., Hong N., Ito H., Kawagoshi Y. (2020). Efficient transition from partial nitritation to partial nitritation/Anammox in a membrane bioreactor with activated sludge as the sole seed source. Chemosphere.

[17] Jin C., Xing J., Chen Z., Meng Y., Fan F., Ahmed T., Meng F. (2021). Development of a Flow-through Biofilm Reactor for Anammox Startup and Operation: Nitrogen Removal and Metacommunity. ACS ES&T Water.

[18] Zhang J., Miao Y., Zhang Q., Sun Y., Wu L., Peng Y. (2020). Mechanism of stable sewage nitrogen removal in a partial nitrification-anammox biofilm system at low temperatures: Microbial community and EPS analysis. Bioresour. Technol..

[19] Awata T., Goto Y., Kindaichi T., Ozaki N., Ohashi A. (2015). Nitrogen removal using an anammox membrane bioreactor at low temperature. Water Sci. Technol..

[20] Teng J., Wu M., Chen J., Lin H., He Y. (2020). Different fouling propensities of loosely and tightly bound extracellular polymeric substances (EPSs) and the related fouling mechanisms in a membrane bioreactor. Chemosphere.

[21] Li X.Y., Yang S.F. (2007). Influence of loosely bound extracellular polymeric substances (EPS) on the flocculation, sedimentation and dewaterability of activated sludge. Water Res..

[22] Lowry O., Rosebrough N., Farr A.L., Randall R. (1951). Protein Measurement with the Folin Phenol Reagent. J. Biol. Chem..

[23] DuBois M., Gilles K.A., Hamilton J.K., Rebers P.A., Smith F. (1956). Colorimetric Method for Determination of Sugars and Related Substances. Anal. Chem..

[24] Rice E.W., Bridgewater L., American Public Health Association (2012). Standard Methods for the Examination of Water and Wastewater.

[25] Gao D.-W., Wang X.-L., Xing M. (2014). Dynamic variation of microbial metabolites and community involved in membrane fouling in A/O-MBR. J. Membr. Sci..

[26] Meng F., Zhang S., Oh Y., Zhou Z., Shin H.S., Chae S.R. (2017). Fouling in membrane bioreactors: An updated review. Water Res..

[27] Neemann F., Rosenberger S., Jefferson B., McAdam E.J. (2013). Non-covalent protein–polysaccharide interactions and their influence on membrane fouling. J. Membr. Sci..

[28] Wei L.L., Wang K., Zhao Q.L., Jiang J.Q., Kong X.J., Lee D.J. (2012). Fractional, biodegradable and spectral characteristics of extracted and fractionated sludge extracellular polymeric substances. Water Res..

[29] Zhou T., Guo J., Liu Q., Liu Y., Wu W., Wang Y., Zhang S., Peng Y. (2024). DOM and DON transformation in full-scale wastewater treatment plants: Comparison of autotrophic and heterotrophic nitrogen removal units. Chem. Eng. J..

[30] Stedmon C.A., Markager S. (2005). Resolving the variability in dissolved organic matter fluorescence in a temperate estuary and its catchment using PARAFAC analysis. Limnol. Oceanogr..

[31] Wu D., Li G.F., Shi Z.J., Zhang Q., Huang B.C., Fan N.S., Jin R.C. (2019). Co-inhibition of salinity and Ni(II) in the anammox-UASB reactor. Sci. Total Environ..

[32] Shen Y., Zhao W., Xiao K., Huang X. (2010). A systematic insight into fouling propensity of soluble microbial products in membrane bioreactors based on hydrophobic interaction and size exclusion. J. Membr. Sci..

[33] Zhang L., Graham N., Derlon N., Tang Y., Siddique M.S., Xu L., Yu W. (2021). Biofouling by ultra-low pressure filtration of surface water: The paramount role of initial available biopolymers. J. Membr. Sci..

[34] Pivokonsky M., Naceradska J., Brabenec T., Novotna K., Baresova M., Janda V. (2015). The impact of interactions between algal organic matter and humic substances on coagulation. Water Res..

[35] Wang Z., Cao J., Meng F. (2015). Interactions between protein-like and humic-like components in dissolved organic matter revealed by fluorescence quenching. Water Res..

[36] Teng J., Shen L., Xu Y., Chen Y., Wu X.L., He Y., Chen J., Lin H. (2020). Effects of molecular weight distribution of soluble microbial products (SMPs) on membrane fouling in a membrane bioreactor (MBR): Novel mechanistic insights. Chemosphere.

[37] Jiang C., Tang X., Feng F., Zhao J., Liu Z., Qu C., Adhikary K.K., Wu D., Tang C.J. (2022). Distinct membrane fouling characteristics of anammox MBR with low NO_2_^−^-N/NH_4_^+^-N ratio. Sci. Total Environ..

[38] Jacquin C., Gambier N., Lesage G., Heran M. (2018). New insight into fate and fouling behavior of bulk Dissolved Organic Matter (DOM) in a full-scale membrane bioreactor for domestic wastewater treatment. J. Water Process Eng..

[39] Luo Y., Liu C., Li C., Shan Y., Mehmood T. (2022). Transformation mechanism and fate of dissolved organic nitrogen (DON) in a full-scale drinking water treatment. J. Environ. Sci..

[40] Yuan Y., Gao J., Wang Z., Zeng L., Xu H., Fu X., Zhao Y. (2023). Deciphering responses of sulfur autotrophic denitrification system to the single and joint stress of Zn(II) and dialkyldimethyl ammonium compounds: Performance, microbial community and different fractions of resistance genes. Chem. Eng. J..

[41] Hong H.C., Peng W., Zhang M.J., Chen J.R., He Y.M., Wang F.Y., Weng X.X., Yu H.Y., Lin H.J. (2013). Thermodynamic analysis of membrane fouling in a submerged membrane bioreactor and its implications. Bioresour. Technol..

[42] Jiang T., Kennedy M.D., de Schepper V., Nam S.N., Nopens I., Vanrolleghem P.A., Amy G. (2010). Characterization of Soluble Microbial Products and Their Fouling Impacts in Membrane Bioreactors. Environ. Sci. Technol..

[43] Wang F., Bian W., Liu W., Liu S., Cui Q., Ai S., Bian D. (2022). Enrichment of functional microorganisms in AAO segmental influent-biofilm filler coupling process to improve the pollutants removal efficiency at low temperature. J. Water Process Eng..

[44] Zhou M., Shi Q., Wang Y. (2022). Application of hydrophilic modified nylon fabric membrane in an anammox-membrane bioreactor: Performance and fouling characteristics. Environ. Sci. Pollut. Res. Int..

[45] Awata T., Goto Y., Kuratsuka H., Aoi Y., Ozaki N., Ohashi A., Kindaichi T. (2021). Reactor performance and microbial community structure of single-stage partial nitritation anammox membrane bioreactors inoculated with Brocadia and Scalindua enrichment cultures. Biochem. Eng. J..

[46] Xu H., Yang X.-L., Liu Y., Xia Y.-G., Song H.-L. (2023). Towards bio-utilization and energy recovery potential exploration of membrane foulant from membrane bioreactor by using microbial fuel cell-centered technology. Bioresour. Technol..

[47] Liu Y., Zhang H., Jiang C., Jiang X., Sakamaki T., Li X. (2023). Effect of bio-electrochemical systems on the removal of organic and inorganic membrane fouling from anaerobic membrane bioreactors. Sep. Purif. Technol..

[48] Ya T., Huang Y., Wang K., Wang J., Liu J., Hai R., Zhang T., Wang X. (2023). Functional stability correlates with dynamic microbial networks in anammox process. Bioresour. Technol..

[49] Cheng B., Wang Y., Zhang D., Wu D., Zan F., Ma J., Miao L., Wang Z., Chen G., Guo G. (2023). Thiosulfate pretreatment enhancing short-chain fatty acids production from anaerobic fermentation of waste activated sludge: Performance, metabolic activity and microbial community. Water Res..

[50] Zhang L., Hao S., Wang Y., Lan S., Dou Q., Peng Y. (2021). Rapid start-up strategy of partial denitrification and microbially driven mechanism of nitrite accumulation mediated by dissolved organic matter. Bioresour. Technol..

[51] Liu S., Cai C., Sun F., Ma M., An T., Chen C. (2024). Advanced nitrogen removal of landfill leachate treatment with anammox process: A critical review. J. Water Process Eng..

