# Supplementary material for: Typical Heterotrophic and Autotrophic Nitrogen Removal Process Coupled with Membrane Bioreactor: Comparison of Fouling Behavior and Characterization
Source: Membranes (Basel). 2024 Oct 7;14(10):214. doi: 10.3390/membranes14100214 (PMC11509564; doi:10.3390/membranes14100214)
Supplement: Supplementary file 1 [file membranes-14-00214-s001.zip › membranes-3018232-supplementary.pdf]

Supplementary data for

Typical heterotrophic and autotrophic nitrogen removal process coupled with MBR: membrane fouling discrepancy and comparison

Qiushan Liu, Tong Zhou, Yuru Liu, Wenjun Wu, Yufei Wang, Guohan Liu, Na Wei, Guangshuo Yin, Jin Guo*

National Engineering Laboratory for Advanced Municipal Wastewater Treatment and Reuse Technology, Beijing University of Technology, Ping leyuan No.100, Beijing 100124, China

List of Supplementary data

Text 1 Dead-end membrane filtration experiment

Table S1 Ingredient of the synthetic wastewater for AO-MBR and PNA-MBR

Table S2 Relative contents of the protein secondary structures of suspended sludge and cake sludge in AO-MBR and PNA-MBR

Fig. S1 Schematic diagram of the AO-MBR (a) and PNA-MBR (b)

Fig. S2 The schematic diagram of the dead-end filtration experiment

Fig. S3 SEM of virgin membrane (a), membrane surface of AO-MBR (c) and PNA-MBR

Fig. S4 Normalized flux evolution of activated sludge mixture from AO-MBR (a) and PNA-MBR (b)

Fig. S5 EEM analysis result of supernatant (a, b) and MBR effluent (c, d) of AO-MBR (a, c) and PNA-MBR (b, d)

Fig. S6 Membrane resistance of SMP and EPS extracted from AO-MBR and PNA-MBR

Fig. S7 FTIR analysis result of SMP, LB-EPS and TB-EPS in active sludge (a, b) and cake sludge (c, d) of AO-MBR (a, c) and PNA-MBR (b, d), respectively

Fig. S8 The second derivative resolution-enhanced curve-fitted amide I region (1700–1600 cm^{-1}) of proteins in the active sludge of AO-MBR (a-c) and PNA-MBR (d-f)

Fig. S9 The second derivative resolution-enhanced curve-fitted amide I region (1700–1600 cm^{-1}) of proteins in cake sludge of AO-MBR (a-c) and PNA-MBR (d-f)

Fig. S10 Molecular weight distribution of SMP, LB-EPS and TB-EPS in suspended sludge (a, c) and the cake sludges (b, d) of AO-MBR and PNA-MBR

Fig. S11 Differences in the phylum level between the suspended sludge (a) and cake sludge (b) in

AO-MBR and PNA-MBR determined by the high-throughput sequencing based 16S rRNA genes

Fig. S12 Differences of active sludge and cake sludge in the phylum level between AO-MBR (a) and PNA-MBR (b) determined by the high-throughput sequencing based 16S rRNA genes

Fig. S13 Differences of active sludge and cake sludge in the genus level between AO-MBR (a) and PNA-MBR (b) determined by the high-throughput sequencing based 16S rRNA genes

Fig. S14 Differences in the genus level between the suspended sludge (a) and cake sludge (b) in AO-MBR and PNA-MBR determined by the high-throughput sequencing based 16S rRNA genes

Text 1 Dead-end membrane filtration experiment

Initially, the system was filtered with 300 mL of Milli-Q water through a virgin membrane to determine stable membrane flux (J_0) at 1bar. Then, the permeate flux of water sample was measured by constant pressure of 1bar nitrogen gas, and the relative flux was determined as J/J_0 (J and J_0 represent the membrane flux at a single moment and the initial flux, respectively). The specific flux decline curve was obtained by plotting the relative flux (J/J_0) versus the filtration volume. The dead-end filtration

The membrane flux was calculated via the following equation (Eq. (1))

$$J = \frac{\Delta V}{A \times \Delta T} \tag{1}$$

where J is the permeate flux ($L/(m^2 \cdot h)$), ΔV is the volume of the permeate (L), A is the membrane effective area (m^2), Δt is the filtration time (h).

Meanwhile, the membrane fouling resistance, including the total resistance (Rt), the intrinsic membrane resistance (Rm), the reversible membrane resistance (Rr) and the irreversible membrane resistance (Rir), was analyzed by Darcy's equation which was elaborated according to the following equation (Eq. (2)-(6))

$$J = \frac{\Delta p}{\mu \times R_t} \tag{2}$$

$$R_t = R_m + R_r + R_{ir} \tag{3}$$

Where J is the permeate flux ($L/(m^2 \cdot h)$), Δp is the transmembrane pressure (Pa), and μ is the dynamic viscosity (Pa •s).

$$R_m = \frac{\Delta p}{\mu \times J_0} \tag{4}$$

$$R_r = \frac{\Delta p}{\mu \times J_1} - \frac{\Delta p}{\mu \times J_0} \tag{5}$$

$$R_{ir} = \frac{\Delta p}{\mu \times J_2} - \frac{\Delta p}{\mu \times J_1} \tag{6}$$

At the end of filtration, the membrane flux was recorded as J_1. The contaminated membrane was backwashed for 10min with Ultra-pure water, and the membrane was re-filtered with 300 mL Ultra-pure water to obtain a stable membrane flux as J_2.

Table S1

Ingredient of the synthetic wastewater for AO-MBR and PNA-MBR.

Main nutrients	Concentration in AO-MBR ($g \cdot L^{-1}$)	Concentration in PNA-MBR ($g \cdot L^{-1}$)
NH_4^+-N [NH_4Cl]	100 mg N L^{-1}	100 mg N L^{-1}
$NaHCO_3$	1	**4**
KH_2PO_4	0.02	0.02
$CaCl_2 \cdot 2H_2O$	0.18	0.18
$MgSO_4 \cdot 7H_2O$	0.3	0.3
COD	**0.5**	0
Na_2EDTA [a]	20	20
$FeSO_4$ [a]	5	5
$ZnSO_4 \cdot 7H_2O$ [a]	0.43	0.43
$CoCl_2 \cdot 6H_2O$ [a]	0.24	0.24
$MnCl_2 \cdot 4H_2O$ [a]	0.99	0.99
$CuSO_4 \cdot 5H_2O$ [a]	0.25	0.25
$NaMoO_4 \cdot 2H_2O$ [a]	0.22	0.22
$NiCl_2 \cdot 6H_2O$ [a]	0.19	0.19
$NaWO_4 \cdot 2H_2O$ [a]	0.014	0.014
H_3BO_4 [a]	0.014	0.014

[a] The trace element solution was also added to 1 $mL \cdot L^{-1}$.

Table S2

Relative contents of the protein secondary structures of suspended sludge and cake sludge in AO-MBR and PNA-MBR.

	**α-helix (1650-1660 cm^{-1})**	**β-sheet (1610-1642,1680-1695 cm^{-1})**	**β-turn (1660-1680 cm^{-1})**	**random coil (1642-1650 cm^{-1})**	**α-helix/(β-sheet+ random coil)**
AO-SMP	17.3±0.67%	51.6±1.68%	17.3±2.83%	11.2±0.81%	0.28
AO-LB	16.1±1.38%	55.9±3.83%	13.7±0.17%	11.5±0.48%	0.24
AO-TB	15.7±2.16%	55.8±0.28%	14.4±1.39%	11.2±2.71%	0.23
PNA-SMP	16.3±0.48%	55.4±1.57%	14.6±2.14%	11.1±0.63%	0.25
PNA-LB	12.6±1.45%	62.1±3.61%	11.7±1.27%	10.7±0.97%	0.17
PNA-TB	11.2±1.57%	65.3±1.39%	9.2±0.49%	10.6±1.26%	0.15
AO-M-SMP	22.06±3.68%	42.75±0.51%	23.86±1.46%	10.26±1.79%	0.42
AO-M-LB	19.37±0.28%	41.46±2.68%	27.13±1.61%	10.49±0.92%	0.37
AO-M-TB	17.80±2.96%	46.80±3.55%	24.30±1.84%	9.79±0.28%	0.31
PNA-M-SMP	17.20±1.06%	52.32±1.57%	19.22±0.43%	11.27±0.84%	0.27
PNA-M-LB	11.18±1.68%	59.89±2.62%	14.55±0.91%	12.21±0.26%	0.16
PNA-M-TB	10.60±0.17%	57.01±2.48%	17.71±1.46%	13.58±0.48%	0.15

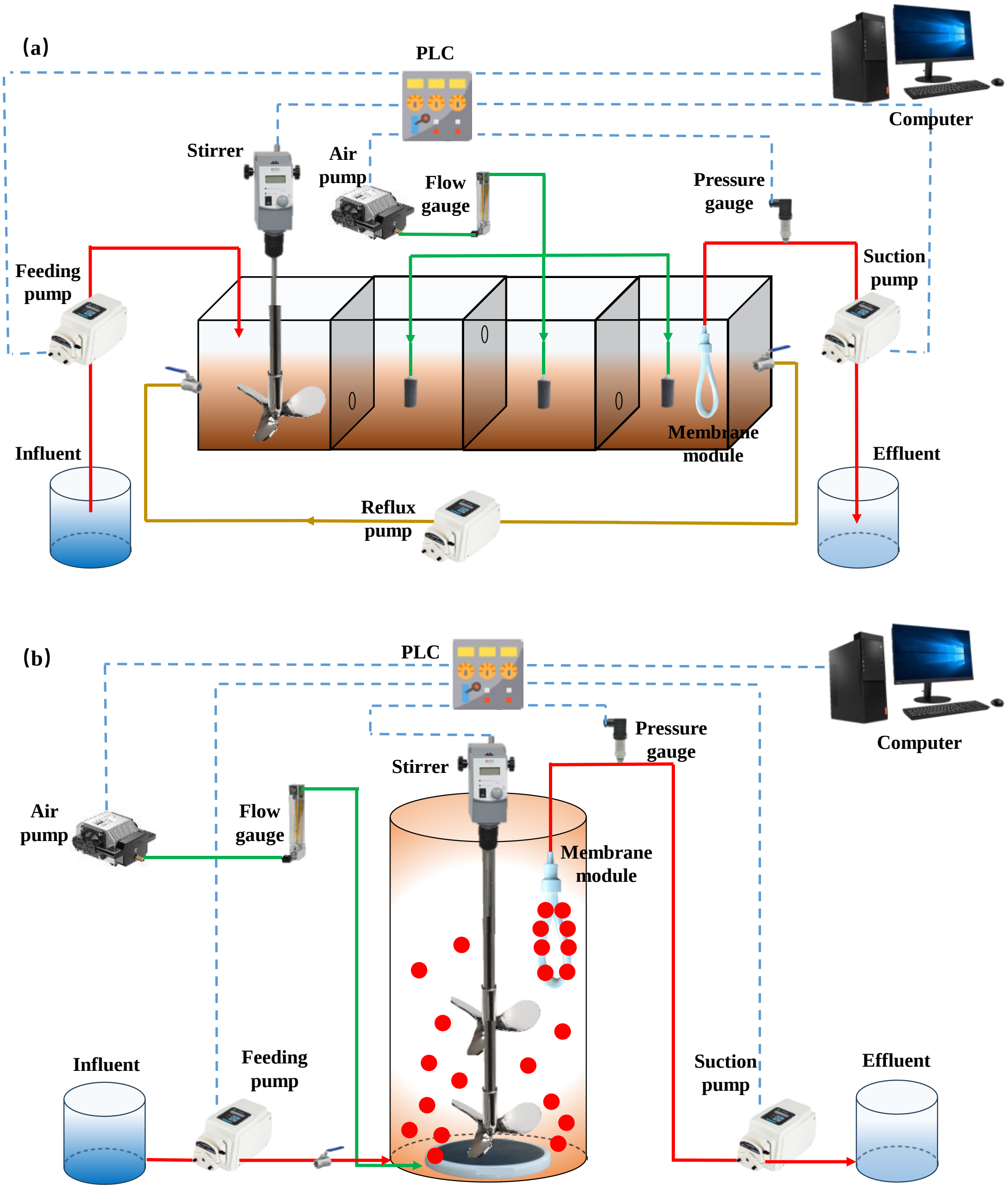

Fig. S1 Schematic diagram of the AO-MBR (a) and PNA-MBR (b).

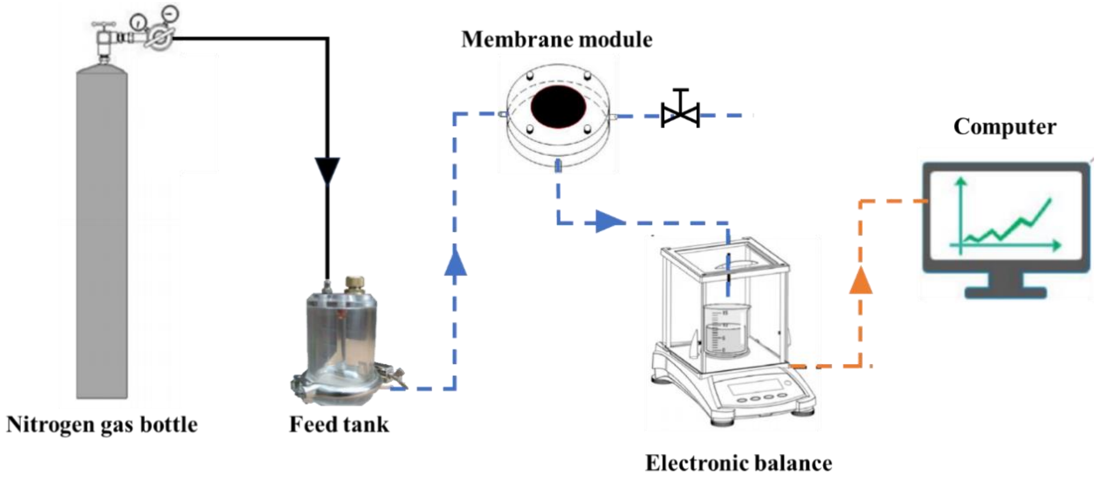

Fig. S2 The schematic diagram of the dead-end filtration experiment.

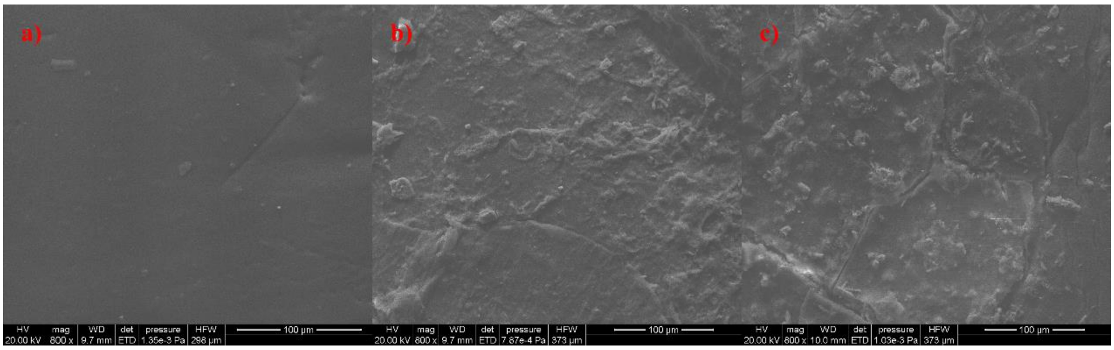

Fig. S3 SEM of virgin membrane (a), membrane surface of AO-MBR (c) and PNA-MBR.

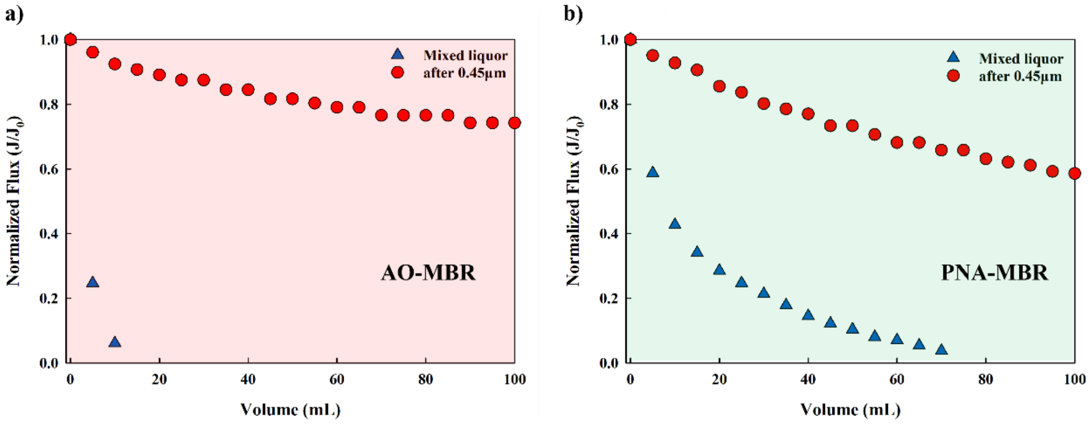

Fig. S4 Normalized flux evolution of activated sludge mixture from AO-MBR (a) and PNA-MBR (b).

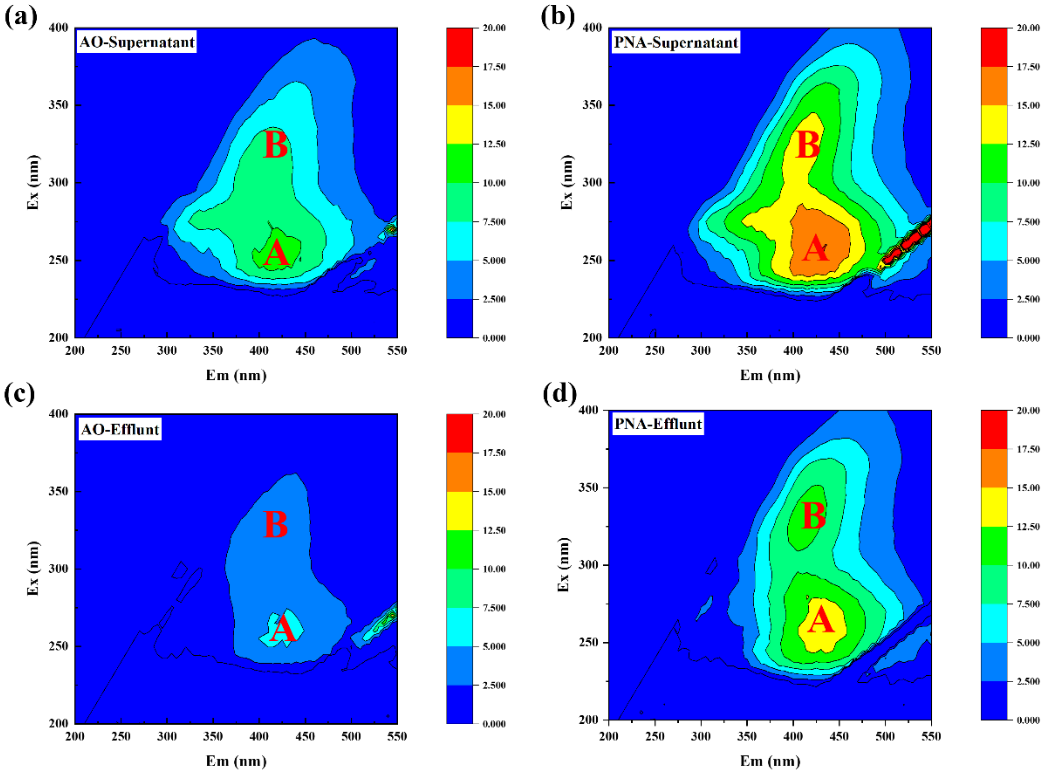

Fig. S5 EEM analysis result of supernatant (a, b) and MBR effluent (c, d) of AO-MBR (a, c) and PNA-MBR (b, d).

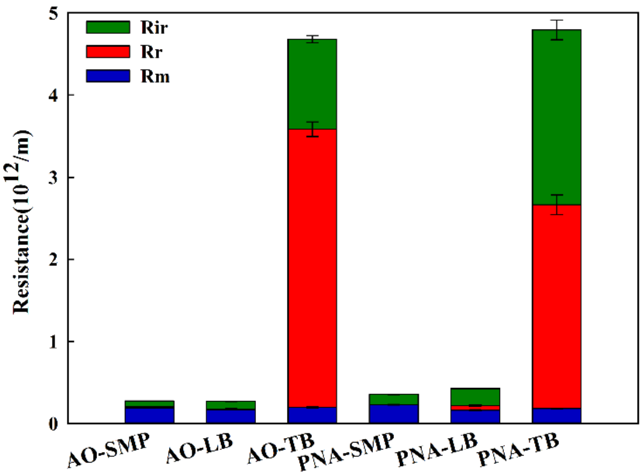

Fig. S6 Membrane resistance of SMP and EPS extracted from AO-MBR and PNA-MBR

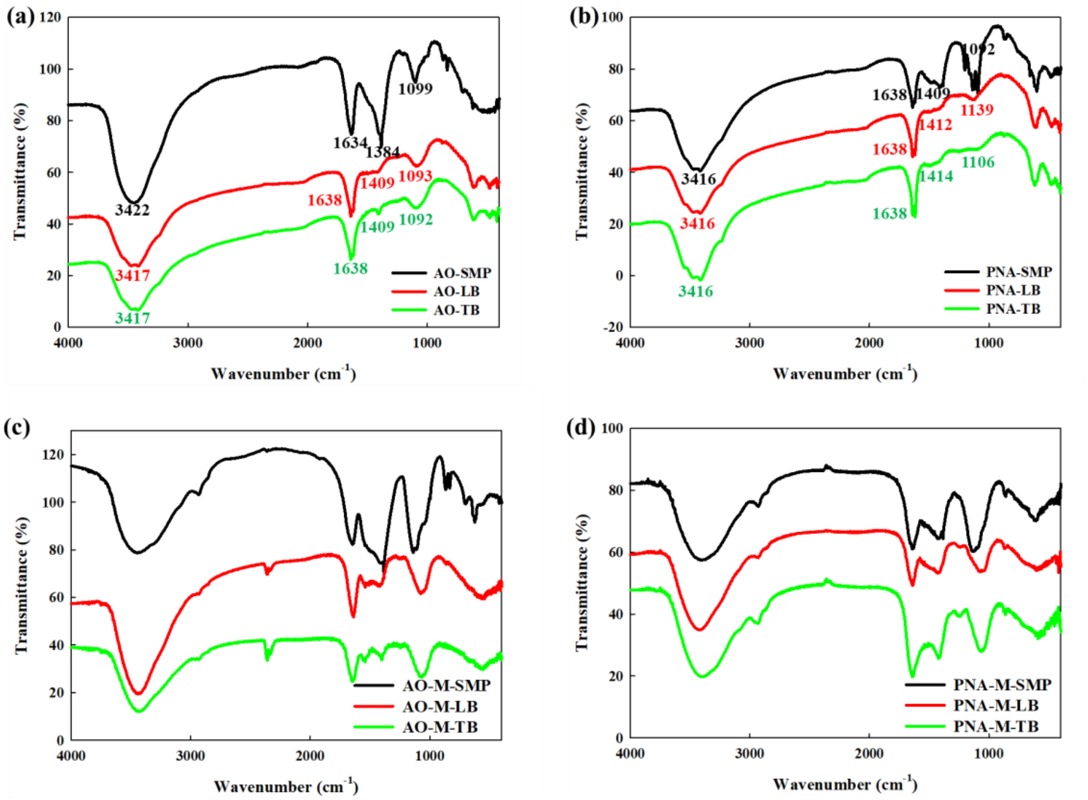

Fig. S7 FTIR analysis result of SMP, LB-EPS and TB-EPS in active sludge (a, b) and cake sludge (c, d) of AO-MBR (a, c) and PNA-MBR (b, d).

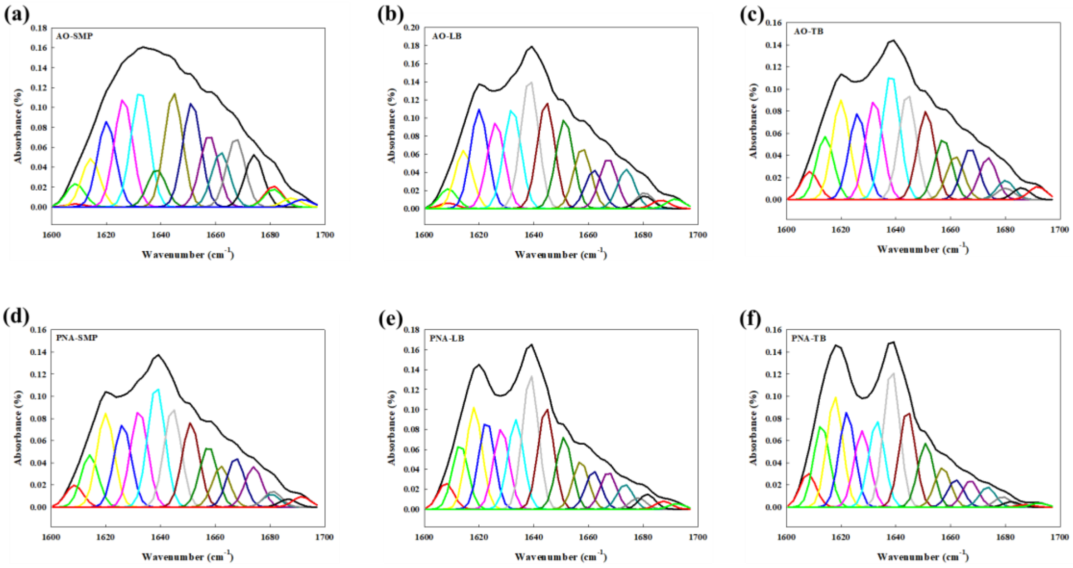

Fig. S8 The second derivative resolution-enhanced curve-fitted amide I region (1700–1600 cm^{-1}) of proteins in the active sludge of AO-MBR (a-c) and PNA-MBR (d-f).

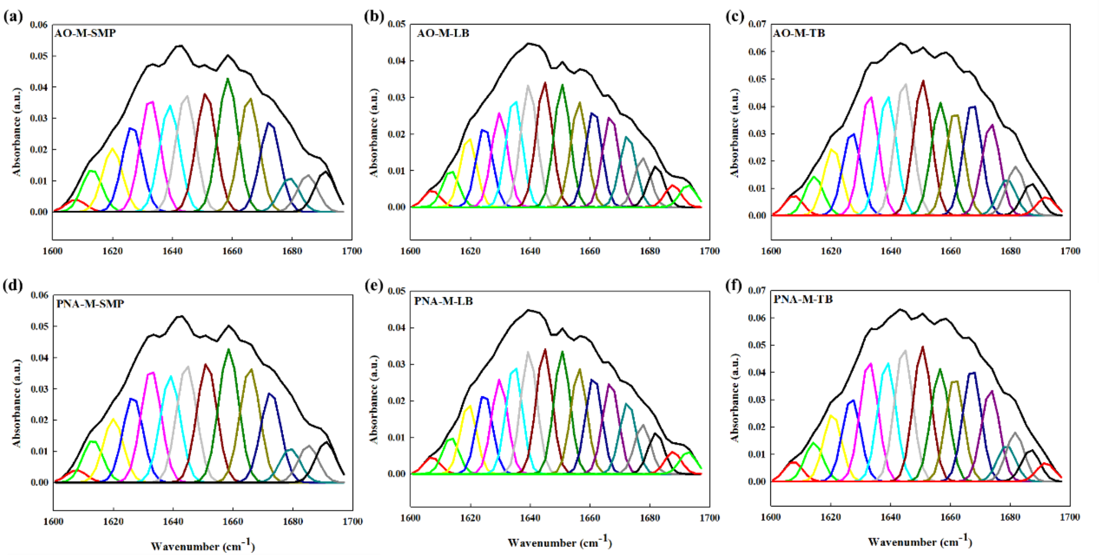

Fig. S9 The second derivative resolution-enhanced curve-fitted amide I region (1700–1600 cm^{-1}) of proteins in cake sludge of AO-MBR (a-c) and PNA-MBR (d-f).

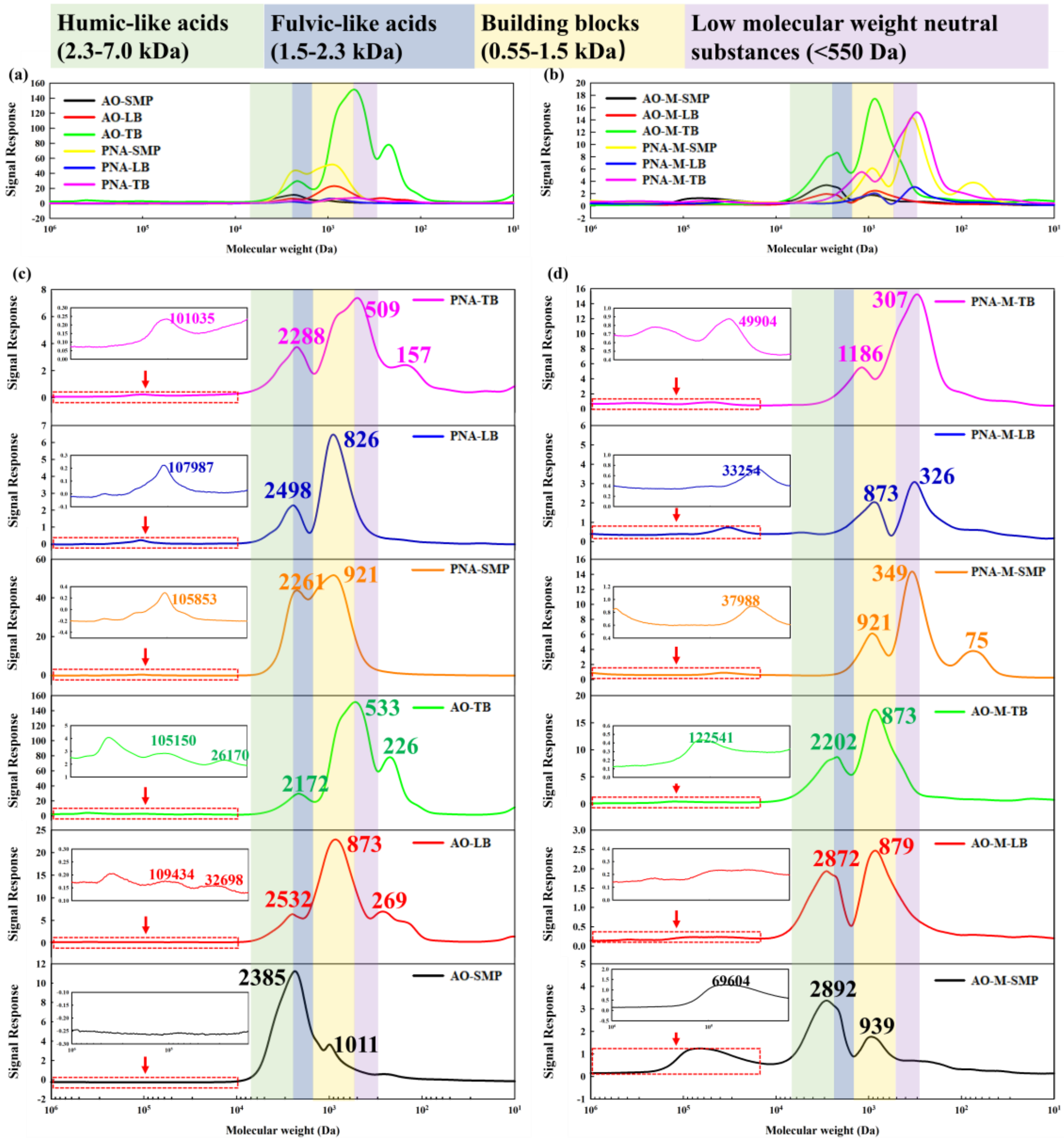

Fig. S10 Molecular weight distribution of SMP, LB-EPS and TB-EPS in suspended sludge (a, c) and the cake sludges (b, d) of AO-MBR and PNA-MBR.

a)

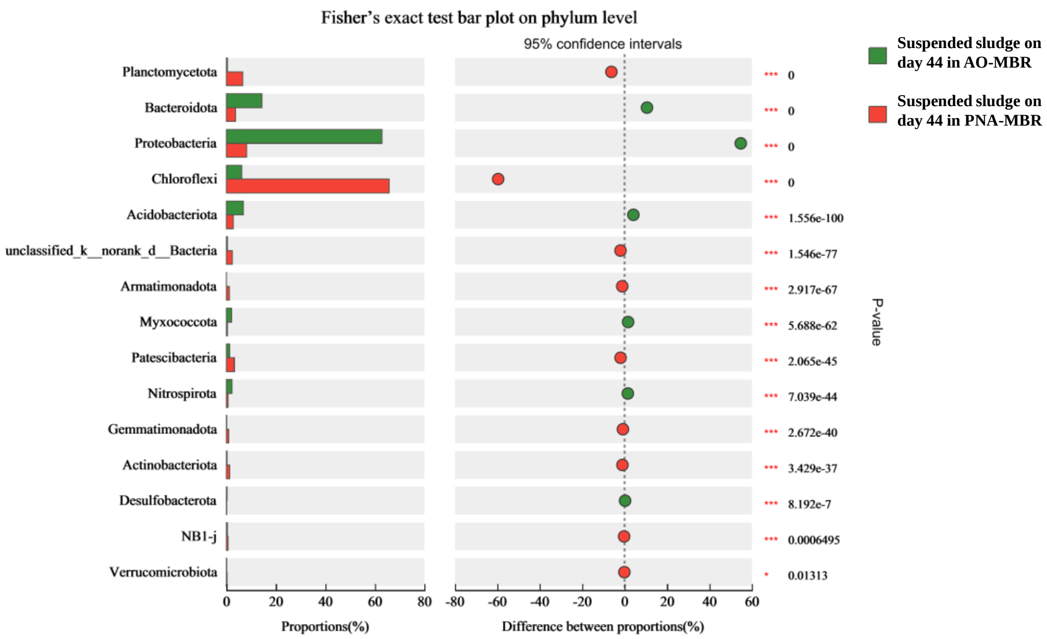

b)

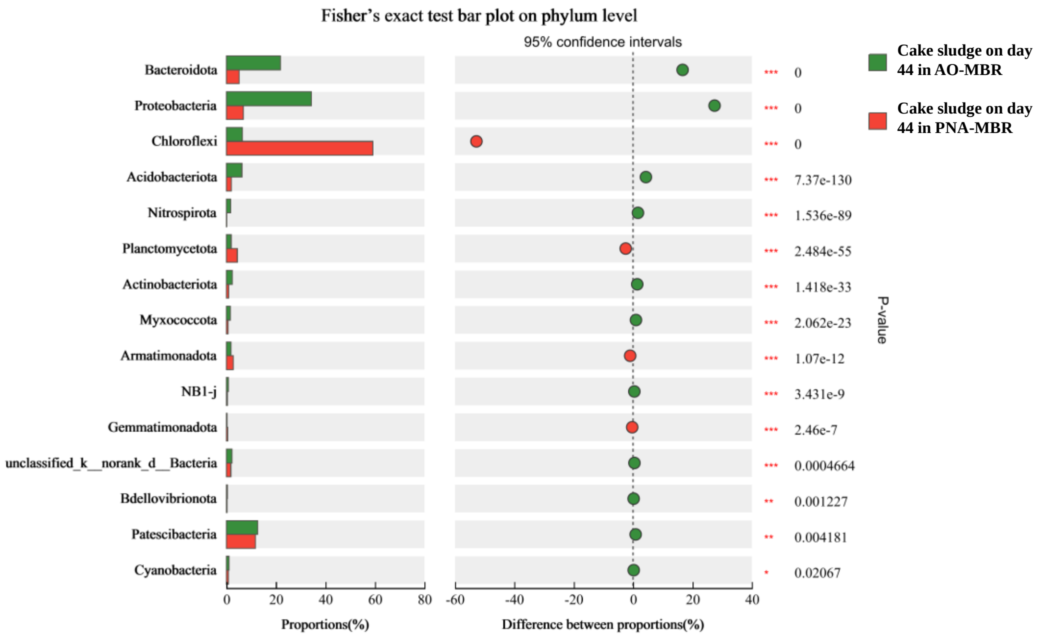

Fig. S11 Differences in the phylum level between the suspended sludge (a) and cake sludge (b) in AO-MBR and PNA-MBR determined by the high-throughput sequencing based 16S rRNA genes (* refers to $p < 0.001$).**

a)

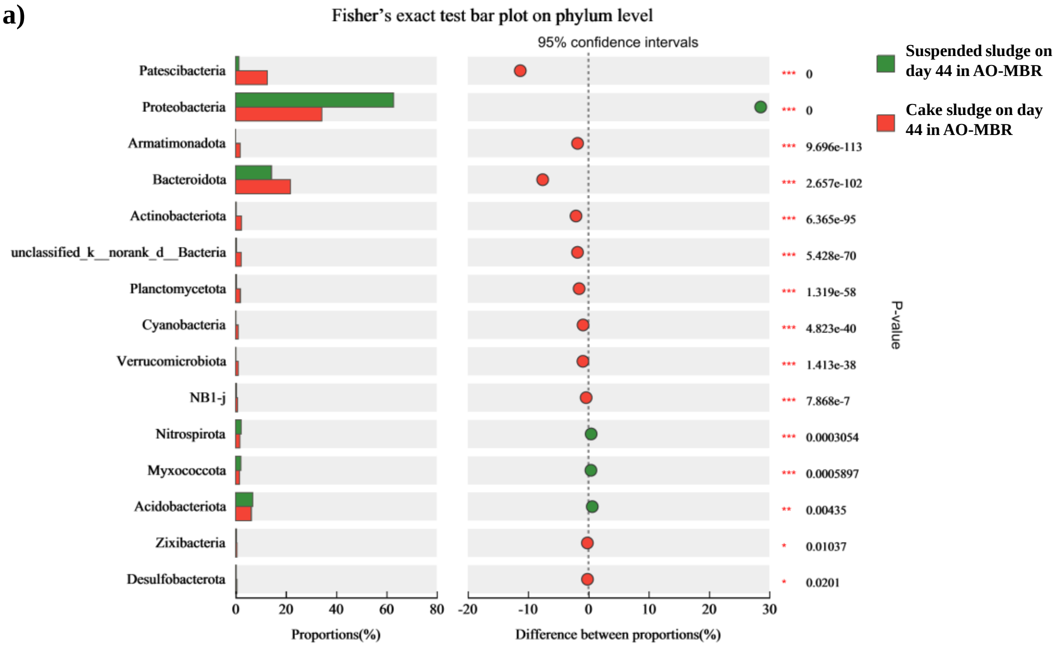

b)

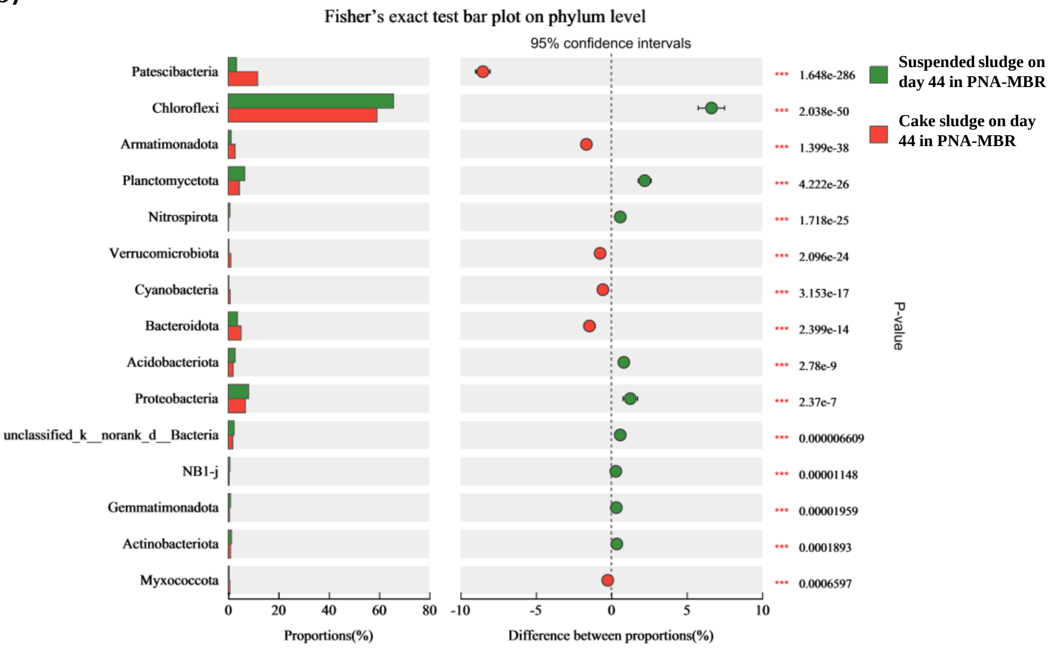

Fig. S12 Differences of active sludge and cake sludge in the phylum level between AO-MBR (a) and PNA-MBR (b) determined by the high-throughput sequencing based 16S rRNA genes (* refers to p < 0.001).**

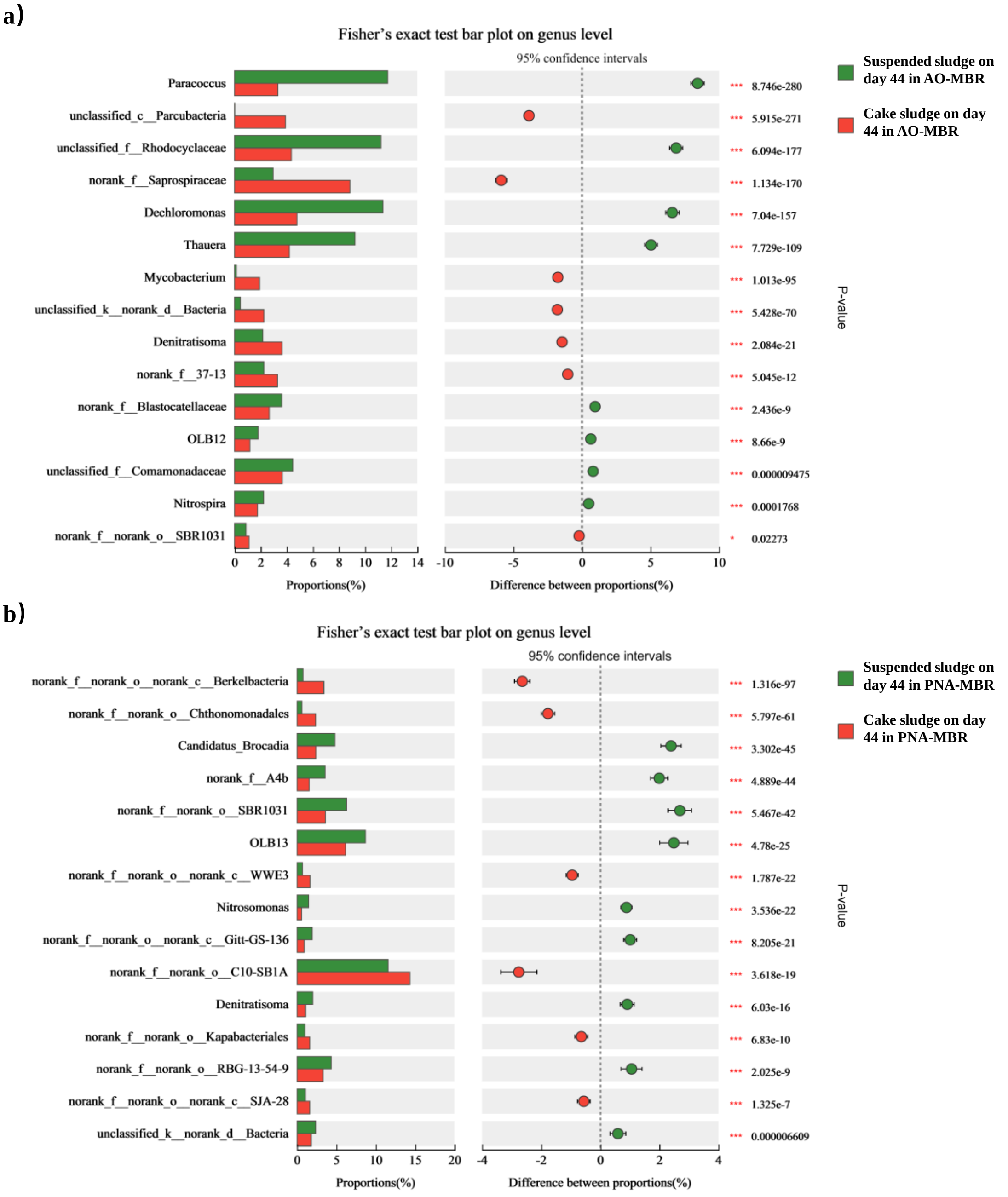

Fig. S13 Differences of active sludge and cake sludge in the genus level between AO-MBR (a) and PNA-MBR (b) determined by the high-throughput sequencing based 16S rRNA genes (* refers to p < 0.001).**

a)

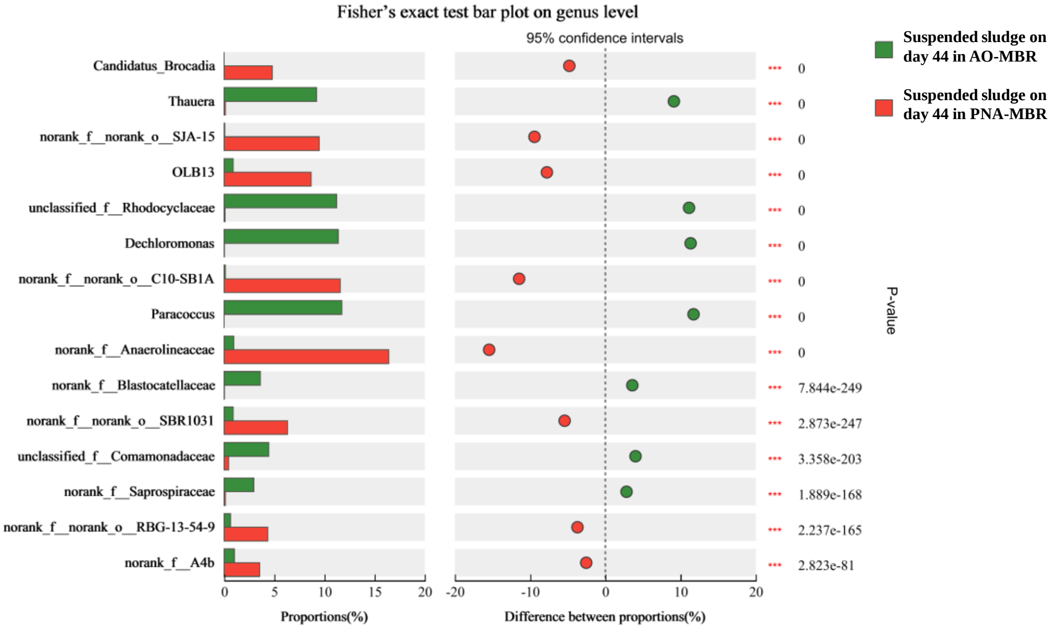

b)

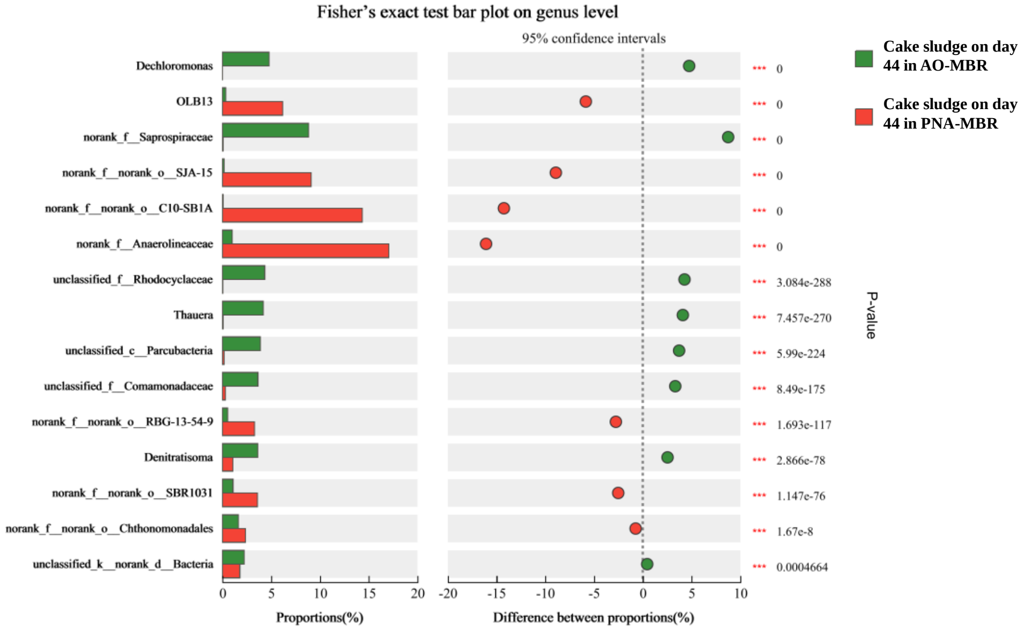

Fig. S14 Differences in the genus level between the suspended sludge (a) and cake sludge (b) in AO-MBR and PNA-MBR determined by the high-throughput sequencing based 16S rRNA genes (* refers to $p < 0.001$).**
